# Internet-delivered cognitive behavioural therapy programme to reduce depressive symptoms in patients with multiple sclerosis: a multicentre, randomised, controlled, phase 3 trial

**DOI:** 10.1016/S2589-7500(23)00109-7

**Published:** 2023-10

**Authors:** Stefan M Gold, Tim Friede, Björn Meyer, Rona Moss-Morris, Joanna Hudson, Susanna Asseyer, Judith Bellmann-Strobl, Andreas Leisdon, Leonie Ißels, Kristin Ritter, David Schymainski, Hayley Pomeroy, Sharon G Lynch, Julia S Cozart, Joan Thelen, Cristina A F Román, Margaret Cadden, Erin Guty, Stephanie Lau, Jana Pöttgen, Caren Ramien, Susan Seddiq-Zai, Anna-Maria Kloidt, Johannes Wieditz, Iris-Katharina Penner, Friedemann Paul, Nancy L Sicotte, Jared M Bruce, Peter A Arnett, Christoph Heesen

**Affiliations:** Klinik für Psychiatrie und Psychotherapie, Campus Benjamin Franklin, Charité–Universitätsmedizin Berlin, Freie Universität Berlin, Humboldt-Universität zu Berlin, and Berlin Institute of Health, Berlin, Germany; Medizinische Klinik mS Psychosomatik, Charité–Universitätsmedizin Berlin, Freie Universität Berlin, Humboldt-Universität zu Berlin, and Berlin Institute of Health, Berlin, Germany; German Center for Mental Health (DZPG), Berlin, Germany; Institut für Neuroimmunologie und Multiple Sklerose, Universitätklinikum Hamburg-Eppendorf, Hamburg, Germany; Department of Medical Statistics, University Medical Center Göttingen, Göttingen, Germany; Research Department, GAIA AG, Hamburg, Germany; Psychology Department, Institute of Psychiatry, Psychology, and Neuroscience, King’s College London, London, UK; Psychology Department, Institute of Psychiatry, Psychology, and Neuroscience, King’s College London, London, UK; NeuroCure Clinical Research Center, Charité–Universitätsmedizin Berlin, Freie Universität Berlin, Humboldt-Universität zu Berlin, and Berlin Institute of Health, Berlin, Germany; NeuroCure Clinical Research Center, Charité–Universitätsmedizin Berlin, Freie Universität Berlin, Humboldt-Universität zu Berlin, and Berlin Institute of Health, Berlin, Germany; Experimental and Clinical Research Center, Max Delbrück Center for Molecular Medicine and Charité–Universitätsmedizin Berlin, Freie Universität Berlin, Humboldt-Universität zu Berlin, and Berlin Institute of Health, Berlin, Germany; Klinik für Psychiatrie und Psychotherapie, Campus Benjamin Franklin, Charité–Universitätsmedizin Berlin, Freie Universität Berlin, Humboldt-Universität zu Berlin, and Berlin Institute of Health, Berlin, Germany; Klinik für Psychiatrie und Psychotherapie, Campus Benjamin Franklin, Charité–Universitätsmedizin Berlin, Freie Universität Berlin, Humboldt-Universität zu Berlin, and Berlin Institute of Health, Berlin, Germany; Klinik für Psychiatrie und Psychotherapie, Campus Benjamin Franklin, Charité–Universitätsmedizin Berlin, Freie Universität Berlin, Humboldt-Universität zu Berlin, and Berlin Institute of Health, Berlin, Germany; Klinik für Psychiatrie und Psychotherapie, Campus Benjamin Franklin, Charité–Universitätsmedizin Berlin, Freie Universität Berlin, Humboldt-Universität zu Berlin, and Berlin Institute of Health, Berlin, Germany; Department of Neurology, Cedars Sinai Medical Center, Los Angeles, CA, USA; Department of Neurology, University of Kansas School of Medicine, Kansas City, KS, USA; Department of Psychology, University of Missouri–Kansas City, Kansas City, MO, USA; Department of Psychology, University of Missouri–Kansas City, Kansas City, MO, USA; Kessler Foundation, Rutgers New Jersey Medical School, Newark, NJ, USA; Department of Physical Medicine & Rehabilitation, Rutgers New Jersey Medical School, Newark, NJ, USA; Department of Neurology, Rutgers New Jersey Medical School, Newark, NJ, USA; Harvard Medical School, Massachusetts General Hospital/Brigham and Women’s Hospital, Boston, MA, USA; Department of Psychology, Penn State University, University Park, PA, USA; Institut für Neuroimmunologie und Multiple Sklerose, Universitätklinikum Hamburg-Eppendorf, Hamburg, Germany; Institut für Neuroimmunologie und Multiple Sklerose, Universitätklinikum Hamburg-Eppendorf, Hamburg, Germany; Institut für Neuroimmunologie und Multiple Sklerose, Universitätklinikum Hamburg-Eppendorf, Hamburg, Germany; Institut für Neuroimmunologie und Multiple Sklerose, Universitätklinikum Hamburg-Eppendorf, Hamburg, Germany; Department of Medical Statistics, University Medical Center Göttingen, Göttingen, Germany; Department of Medical Statistics, University Medical Center Göttingen, Göttingen, Germany; Department of Neurology, Inselspital, Bern University Hospital, University of Bern, Bern, Switzerland; NeuroCure Clinical Research Center, Charité–Universitätsmedizin Berlin, Freie Universität Berlin, Humboldt-Universität zu Berlin, and Berlin Institute of Health, Berlin, Germany; Experimental and Clinical Research Center, Max Delbrück Center for Molecular Medicine and Charité–Universitätsmedizin Berlin, Freie Universität Berlin, Humboldt-Universität zu Berlin, and Berlin Institute of Health, Berlin, Germany; Department of Neurology, Cedars Sinai Medical Center, Los Angeles, CA, USA; Department of Biomedical and Health Informatics, School of Medicine, University of Missouri–Kansas City, Kansas City, MO, USA; Department of Psychology, Penn State University, University Park, PA, USA; Institut für Neuroimmunologie und Multiple Sklerose, Universitätklinikum Hamburg-Eppendorf, Hamburg, Germany

## Abstract

**Background:**

Depression is three to four times more prevalent in patients with neurological and inflammatory disorders than in the general population. For example, in patients with multiple sclerosis, the 12-month prevalence of major depressive disorder is around 25% and it is associated with a lower quality of life, faster disease progression, and higher morbidity and mortality. Despite its clinical relevance, there are few treatment options for depression associated with multiple sclerosis and confirmatory trials are scarce. We aimed to evaluate the safety and efficacy of a multiple sclerosis-specific, internet-based cognitive behavioural therapy (iCBT) programme for the treatment of depressive symptoms associated with the disease.

**Methods:**

This parallel-group, randomised, controlled, phase 3 trial of an iCBT programme to reduce depressive symptoms in patients with multiple sclerosis was carried out at five academic centres with large outpatient care units in Germany and the USA. Patients with a neurologist-confirmed diagnosis of multiple sclerosis and depressive symptoms were randomly assigned (1:1:1; automated assignment, concealed allocation, no stratification, no blocking) to receive treatment as usual plus one of two versions of the iCBT programme Amiria (stand-alone or therapist-guided) or to a control condition, in which participants received treatment as usual and were offered access to the iCBT programme after 6 months. Masking of participants to group assignment between active treatment and control was not possible, although raters were masked to group assignment. The predefined primary endpoint, which was analysed in the intention-to-treat population, was severity of depressive symptoms as measured by the Beck Depression Inventory-II (BDI-II) at week 12 after randomisation. This trial is registered at ClinicalTrials.gov, NCT02740361, and is complete.

**Findings:**

Between May 3, 2017, and Nov 4, 2020, we screened 485 patients for eligibility. 279 participants were enrolled, of whom 101 were allocated to receive stand-alone iCBT, 85 to receive guided iCBT, and 93 to the control condition. The dropout rate at week 12 was 18% (50 participants). Both versions of the iCBT programme significantly reduced depressive symptoms compared with the control group (BDI-II between-group mean differences: control *vs* stand-alone iCBT 6·32 points [95% CI 3·37–9·27], p<0·0001, effect size d=0·97 [95% CI 0·64–1·30]; control *vs* guided iCBT 5·80 points [2·71–8·88], p<0·0001, effect size d=0·96 [0·62–1·30]). Clinically relevant worsening of depressive symptoms was observed in three participants in the control group, one in the stand-alone iCBT group, and none in the guided iCBT group. No occurrences of suicidality were observed during the trial and there were no deaths.

**Interpretation:**

This trial provides evidence for the safety and efficacy of a multiple sclerosis-specific iCBT tool to reduce depressive symptoms in patients with the disease. This remote-access, scalable intervention increases the therapeutic options in this patient group and could help to overcome treatment barriers.

**Funding:**

National Multiple Sclerosis Society (USA).

## Introduction

Compared with the general population, depression is around three to four times more prevalent in patients who have a chronic illness.^1^ In patients with multiple sclerosis, depression is the most common comorbidity,^2^ with a 12-month prevalence of major depressive disorder estimated at approximately 25%^3^ and a lifetime prevalence of up to 50%.^4^ Depression in this population is associated with cognitive impairment^5^ and reduced adherence to disease-modifying therapies.^6^ Depression also contributes substantially to the psychosocial burden of multiple sclerosis.^7^ Notably, depression could be one of the earliest manifestations of multiple sclerosis,^8^ and patients with comorbid depression are at risk of faster disability progression than those without.^9,10^ Depression in multiple sclerosis is also linked to higher cardiovascular morbidity and all-cause mortality,^11,12^ highlighting the effect of this comorbidity.

Despite its clinical importance, therapeutic options for depression in patients with multiple sclerosis remain scarce. Evidence for the efficacy of antidepressants is insufficient.^13,14^ By contrast, there is increasing evidence for the benefits of cognitive behavioural therapy (CBT) from numerous randomised controlled trials, as supported by meta-analyses.^15,16^

However, therapists who can deliver CBT and other psychological treatments that are tailored to the needs of patients with multiple sclerosis are not widely available. Common multiple sclerosis symptoms—such as mobility impairments, fatigue, or cognitive difficulties—can be additional barriers. To overcome these issues, remote-access options—such as CBT delivered via telephone—can be effective for the treatment of depression associated with multiple sclerosis;^17^ however, such interventions still require specialised therapists and are not available at scale. Effective, stand-alone, and scalable remote-access treatment options could therefore substantially improve clinical care. Online psychological interventions could help to reduce symptoms of depression in patients who do not also have a chronic medical illness^18^ as well as in patients with underlying medical conditions, including multiple sclerosis.^19^ Several small, single-centre, randomised controlled trials have been conducted using psychological internet-based tools to treat depression in patients with multiple sclerosis, yielding mixed results.^20–22^ In one of these trials, conducted in Germany, efficacy to reduce depressive symptoms in multiple sclerosis was shown for the unguided, generic internet-based CBT (iCBT) programme known as Deprexis.^20^ However, large confirmatory trials for any treatments of multiple sclerosis-associated depression that could inform clinical practice are scarce. To address this gap, we conducted a multicentre phase 3 trial to test the efficacy of the multiple sclerosis-specific iCBT tool Amiria, developed from the Deprexis programme, to reduce depressive symptoms in patients with multiple sclerosis.

## Methods

### Study design

This was a three-arm, parallel-group, multicentre, randomised, controlled, phase 3 trial conducted at five academic centres in Germany and the USA with large outpatient multiple sclerosis care units (Charité–Universitätsmedizin Berlin, Berlin, Germany; Universitätsklinikum Hamburg-Eppendorf, Hamburg, Germany; Cedars Sinai Medical Center, Los Angeles, CA, USA; Penn State University, University Park, PA, USA; and University of Missouri–Kansas City School of Medicine, Kansas City, KS, USA).

The study was reviewed and approved by the appropriate ethics review boards at each trial site before enrolling their first participant (Charité Institutional Review Board [EA1/102/16]; Ethics Board of the Chamber of Physicians Hamburg [PMC-137/16V]; UMKC Institutional Review Board [16–205]; Penn State University Institutional Review Board [#00004660]; and Cedars-Sinai Medical Center Institutional Review Board [Pro00045146]). The study protocol is available in the appendix (pp 39–67).

### Participants

Inclusion criteria were age at least 18 years; neurologist-confirmed diagnosis of multiple sclerosis according to McDonald criteria;^23^ self-reported depressive symptoms (BDI-Fastscreen>4); fluency in German or English (depending on study site); willingness to engage in self-administration of an iCBT intervention for 12 weeks and complete follow-up; ability to travel to the outpatient centre for two clinical assessments (baseline and week 12); internet access at home; and provision of informed consent. Exclusion criteria were unwillingness or inability to consent; a diagnosis of bipolar disorder or psychosis (as established in the clinical interview); substantial neurocognitive impairments, dementia or autism (based on medical history and clinical judgement by the physician at the recruitment site); moderate or high risk of suicide by clinical impression; very severe depression that would interfere with the ability to participate in the study (based on clinical judgement by the physician at the recruitment site, patients with very severe depression were referred to local psychiatric services for immediate treatment); current psychotherapy or behavioural treatments for depression (defined as regular face-to-face sessions with a qualified psychotherapist, at study intake, either in individual or group settings, at a frequency of at least two sessions per month, started within the past 6 months before study intake); having started pharmacotherapy for depression within the past 2 months; a multiple sclerosis relapse or steroid treatment in the past 4 weeks; concurrent participation in another interventional clinical trial; and refusal to consent to the saving, processing and forwarding of pseudonymised data.

Participants were recruited from the respective outpatient units of the participating centres, via referrals from collaborating neurologists, and through self-referrals from online recruitment announcements in electronic newsletters sent out by the National Multiple Sclerosis Society in the USA and the German Multiple Sclerosis Society during the recruitment period. All participants provided written informed consent before enrolment and were financially compensated for their time and effort of clinical visits and outcome assessments.

### Randomisation and masking

Participants were randomly assigned in a 1:1:1 ratio to one of the three study groups (stand-alone iCBT, guided iCBT, or control) by a fully automated random-allocation sequence built into the study platform (no blocking or stratification). To ensure concealed allocation, eligibility was established and all baseline assessments completed before executing fully automated randomisation via the study platform, in compliance with CONSORT guidelines. After leaving the study centre, participants received an automated email sent from the study platform to their registered email address informing them to log into the platform, where they would find a message regarding their group assignment and instructions to access the multiple sclerosis iCBT programme (if assigned to one of the active groups) or how long they would have to wait until access would be available (if assigned to the control group).

For patient-reported outcomes, masking of participants to group assignment between active treatment and control was not possible. However, for the clinician-reported outcomes (Montgomery-Åsberg Depression Rating Scale [MADRS] and clinical interviews), arrangements were made to keep raters masked to group assignment—participants were contacted to schedule study visits by staff who were not involved in the clinical assessments and participants were specifically instructed not to reveal their assigned group to the examiner during the visits.

### Procedures

All participants were examined by a neurologist to confirm their multiple sclerosis diagnosis and to record relevant clinical information, such as current use of disease-modifying therapies. At baseline and at week 12, we also obtained patient-reported (Patient-Determined Disease Steps [PDDS]^24^) and clinician-rated (Expanded Disability Status Scale [EDSS]^25^) disability scores. Neuropsychological function was examined by a trained rater using the components of the Brief International Cognitive Assessment for Multiple Sclerosis:^26^ the Symbol Digit Modalities Test, the California Verbal Learning Test II, and the Brief Visuospatial Memory Test-Revised.

We used the Amiria iCBT programme either as stand-alone iCBT or with added standardised email support by a clinical psychologist (guided iCBT), and compared the results with those from the control group.

This multiple sclerosis-specific iCBT programme is based on principles and techniques used in CBT. The programme consists of ten sequential modules plus a summary module. Like Deprexis, it uses a simulated-dialogue approach by presenting brief, conversational text passages followed by multiple response options from which users can select. Subsequent content is then tailored to the patient’s individual responses. The user’s responses therefore determine the specific path through each module, and a simulated conversational flow—albeit in text rather than spoken format—is created. Depending on factors such as the user’s reading speed, response choices, or decisions to listen to optional audio recordings, each module can be completed in about 30–60 min. Contents are psychoeducation; behavioural activation; cognitive modification; mindfulness and acceptance; interpersonal skills; relaxation, physical exercise, and lifestyle modification; problem solving; expressive writing and forgiveness; positive psychology; and emotion-focused interventions. The iCBT used in this trial contained several elements specific to multiple sclerosis, which were developed in close collaboration with advisers living with the disease and experienced clinicians including neurologists, neuropsychologists, and psychotherapists with extensive experience in the care of patients with multiple sclerosis (appendix pp 5–14). Detailed descriptions of the content and functionalities of Deprexis, the generic version of this programme from which the multiple sclerosis-specific intervention was developed, can also be found in previous publications.^27,28^

Participants in the guided iCBT group received the multiple sclerosis-specific iCBT programme plus scheduled email contact with a therapist. The basic structure of the email support was based on our previous work^29^ and is described in detail in the manual (appendix pp 15–24). Three therapists with qualifications in clinical psychology or behavioural therapy were responsible for email support in the trial (AL and LI for patients enrolled in the German study sites and JH for patients enrolled in the USA). Supervision by an experienced, licensed psychotherapist (BM) and a registered psychologist with CBT qualifications (RM-M) was provided monthly for the study therapists.

The iCBT programme (both stand-alone and guided versions) tracks several indicators of usage, including days with activity in the programme and the number of modules participants completed. In addition, we tracked minutes with activity—a metric that uses 5-min blocks and excludes each block of inactivity—so that the logged usage times are a good estimate of time actually spent working with the programme.

Participants assigned to the control group continued to receive treatment as usual. After 6 months, participants in this group were offered access to the iCBT programme (unguided version).

In the primary trial phase, patients were randomly assigned to one of the three trial groups for 12 weeks. The trial also included a controlled extension phase (12 weeks–6 months after inclusion), during which participants who were randomly assigned to either stand-alone or guided iCBT were given continued access to the programme and could continue to work with it. The trial further included a non-controlled maintenance phase (6–12 months). At that time, participants who were originally assigned to the control group were offered access to the stand-alone iCBT programme. Participants in the other two groups, who had received guided iCBT and stand-alone iCBT, were randomly assigned to receive access to iCBT booster sessions (additional CBT content that is compatible with but goes beyond content already covered in previous programme modules) or not to receive such booster sessions. The booster sessions provided access to advanced CBT content and exercises as well as continued access to the previous content. A full overview of the trial design is provided in the appendix (p 43).

### Outcomes

The predefined primary endpoint was the total score of the Beck Depression Inventory-II (BDI-II) at week 12 after randomisation (end of the primary trial phase). The BDI-II is a 21-item self-report depression questionnaire that has been found to be reliable, valid, and sensitive for assessing depression in the context of multiple sclerosis^30^ and produces similar results when administered in paper or online formats.^31^ The BDI-II score was obtained during the primary trial phase (baseline and week 12) and the extension phase (month 6 and month 12). During the primary trial phase, the BDI-II was also obtained online via the study platform at two interim time points (after 4 weeks and 8 weeks).

Preregistered secondary endpoints of this trial were patient-reported outcomes of quality of life using a generic questionnaire (the WHO Quality of Life-Brief Version [WHOQOL-BREF],^32^ consisting of four domains) and a multiple sclerosis-specific questionnaire (Multiple Sclerosis Impact Scale-29 [MSIS-29]^33^) and patient-reported outcomes for fatigue (the Fatigue Scale for Motor and Cognitive Functions [FSMC],^34^ with two domains, and the Chalder Fatigue Scale [CFS]^35^). All secondary endpoints were predefined and preregistered as change from baseline to week 12. Additional endpoints included clinician ratings of depressive symptoms (using the MADRS^36^) and caseness of major depressive disorder based on Diagnostic and Statistical Manual of Mental Disorders (DSM) diagnostic criteria. These endpoints were predefined in the trial protocol. We also explored the effect of added therapist support (by comparing stand-alone iCBT and guided iCBT), stability of treatment effects at 6 months post baseline, and the effect of booster sessions versus no booster sessions at month 12 post baseline as additional outcome analyses of interest.

Predefined safety measures focused on new occurrence of suicidal ideation or intent. Self-report data on suicidal ideation and behaviour (from the Suicide Behaviors Questionnaire-Revised [SBQ-R]^37^) and the BDI-II were used (acute suicidality as indicated by response 3a or 3b on SBQ-R item 3 plus a score of 5 or 6 on SBQ-R item 4 or a score of 3 on BDI-II item 9 at any assessment). These responses would automatically be flagged by the study platform and responded to by the responsible study centre staff within 24 h, following the trial’s standard operating procedure for suicidality. Additional predefined safety measures were hospitalisation due to psychiatric disorder classified according to International Classification of Diseases (ICD) or DSM, suicidality detected during the clinical interviews or message exchange with the study therapists, or any lethal or life-threatening event (including suicide or suicide attempt). As an additional post-hoc safety measure, we analysed clinically relevant worsening of depression during the trial defined as change in BDI-II scores from below to above the cutoff for caseness (BDI-II>13).

### Statistical analysis

A sample size of 100 patients per intervention group gives a conjunctive power (probability of rejecting both null hypotheses comparing stand-alone iCBT and guided iCBT to control) of 90% for a Dunnett’s test at the usual one-sided significance level of 2·5% assuming standardised mean differences of 0·5 for stand-alone iCBT versus control and 0·8 for guided iCBT versus control in the primary outcome change in BDI-II from baseline to week 12. The standardised mean difference for the stand-alone iCBT was informed by the effect observed in our previous phase 2 trial^20^ and the minimal clinically important difference of the BDI-II.^38^ On the basis of the dropout rates observed in our previous trial,^20^ the sample size was adjusted for 20% dropout: we aimed to recruit 125 patients per group, resulting in a total sample size of 375 patients. The power was simulated with 10 000 replications using East version 6.3.

The full analysis set was based on intention-to-treat principles: all randomly assigned patients with at least one post-baseline assessment were included in the analysis. A modified intention-to-treat population was analysed in a sensitivity analysis, including all patients who had registered in the iCBT programme. The safety set was defined as all participants who registered in the iCBT programme.

All analyses were predefined in the statistical analysis plan before unmasking (appendix pp 25–38) and follow relevant regulatory guidelines for the statistical analysis of randomised trials, including CPMP/ICH/363/96 E9 on statistical principles for clinical trials and guidance EMA/CHMP/295050/2013 from the European Medicines Agency on adjustment for baseline covariates in clinical trials. In line with these recommendations, all analyses are adjusted for baseline levels of the respective outcome measure.

The primary outcome (change in BDI-II from baseline to week 12) was analysed by means of linear mixed-effects models for repeated measures adjusted for baseline measurements with fixed effects for intervention, region (USA or Germany), time and baseline BDI-II score, and random subject effects for individual patients including all patients with at least one post-baseline measurement.^39^ Least-squares means (with 95% CI) are reported for the intervention groups as well as the difference between the least-squares group means (with 95% CI). Stand-alone iCBT versus control and guided iCBT versus control were assessed by a Dunnett’s test controlling the familywise type I error rate at the level of 2·5% (one-sided). In a secondary step, the added value of therapist email support iCBT versus stand-alone iCBT was tested at a two-sided level of 5%, if the effectiveness of stand-alone iCBT and guided iCBT for reducing depressive symptoms in multiple sclerosis was shown.

Standardised effect sizes are reported as Cohen’s d^40^ with corresponding 95% CIs (R package effsize, version 0.8.1). Effect size variances were computed as previously described.^41^ Pooled effect sizes after multiple imputation were computed on the basis of Rubin’s rules.^42^ As sensitivity analyses, last observation carried forward, multiple imputations, complete case, and analysis of the modified intention-to-treat population were used to deal with missing values for the BDI-II score (missing visits), and for each method an ANCOVA for the BDI-II score after 12 weeks was carried out with BDI-II score at baseline as covariate.

The analyses of secondary endpoints followed the same approach as for the primary endpoint. The number of patients with a clinical diagnosis of current major depressive disorder was analysed using a logistic regression model with the variables treatment group and baseline score of BDI-II. Statistical programming was done by the trial statisticians (TF, A-MK, and JW) using R version 4.0.0 and SAS version 9.4.

An independent data monitoring committee was established before the start of the trial to be consulted in case any predefined safety concerns were registered in the study platform.

This trial is registered at ClinicalTrials.gov, NCT02740361.

### Role of the funding source

The funder of the study had no role in study design, data collection, data analysis, data interpretation, or writing of the report.

## Results

**Figure 1:**
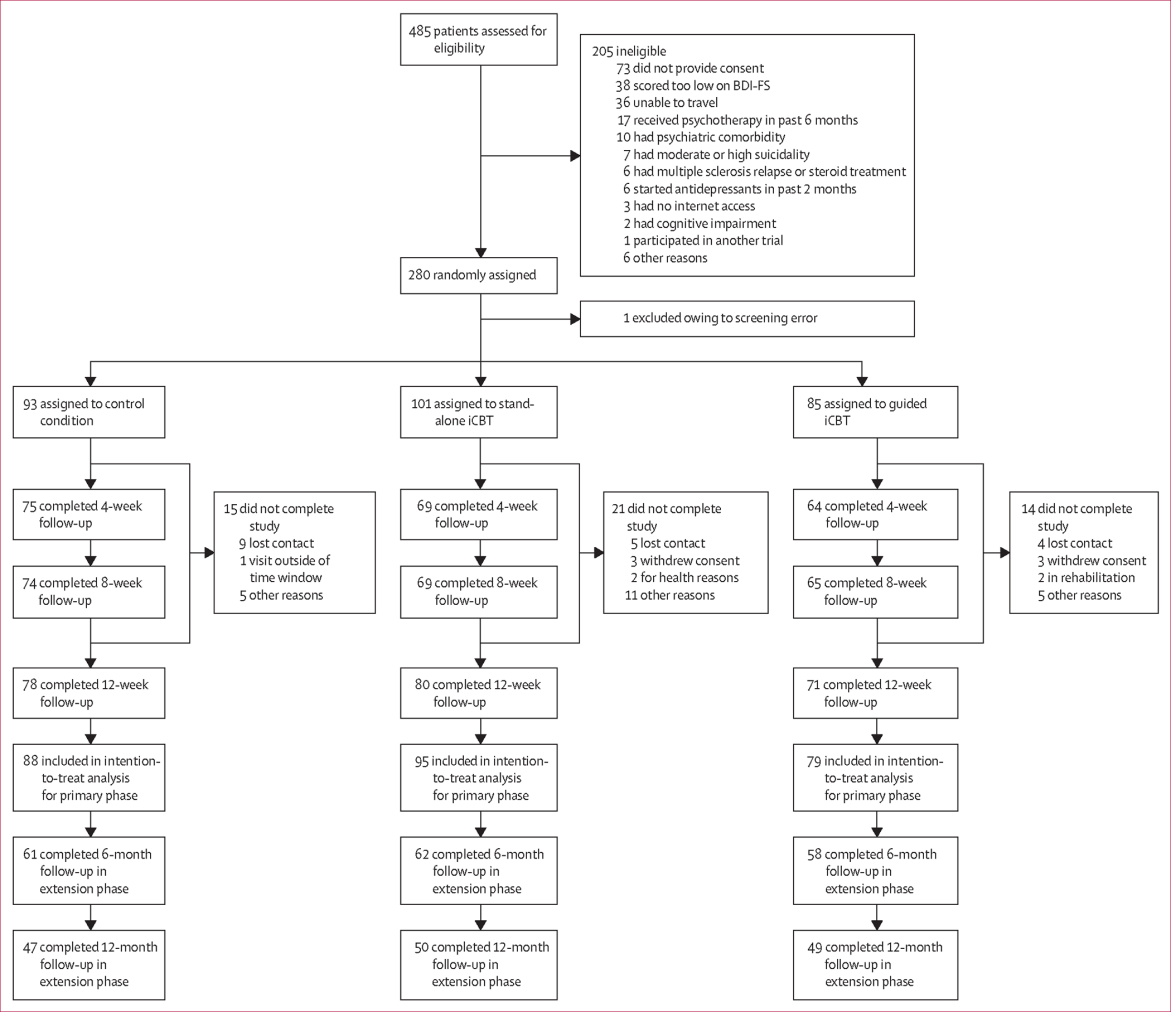
Trial profile The intention-to-treat population included all randomly assigned patients who completed at least one post-baseline assessment (ie, any or all of the 4-week, 8-week, or 12-week follow-up assessments).

Participants were enrolled between May 3, 2017, and Nov 4, 2020; the last patient completed the primary trial phase on Jan 31, 2021. The original target sample size was 375 with a recruitment period of 36 months. Recruitment was temporarily halted owing to the COVID-19 pandemic and remained difficult during 2020 for all centres; the recruitment phase was therefore extended and was stopped in November, 2020, when the available funding was exhausted. We screened 485 patients, of whom 205 were excluded, 280 were enrolled and randomly assigned, and 279 were allocated to the trial groups with one excluded owing to a screening error (Charité Universitätsmedizin Berlin n=35, Universitätsklinikum Hamburg-Eppendorf n=102, Penn State University n=47, University of Missouri n=51, Cedars Sinai Medical Center n=44). The dropout rate, defined as the proportion of participants who did not complete outcome measures, for the primary trial phase (12 weeks) was 18% (50 of 279 participants; figure 1).

Baseline sociodemographic and clinical characteristics are shown in tables 1, 2. 44% of the participants were treated with antidepressants at baseline. PDDS and EDSS scores indicated moderate disability on average, but the sample included a wide range from no or mild disability to severe disability (range 0–8 for both EDSS and PDDS scores).

Participants assigned to the iCBT groups used the programme for a median of 11·0 days (IQR 6·0–18·2) of the 12-week primary trial phase (median for stand-alone iCBT 10·0 [6·0–18·5]; guided iCBT 12·0 [8·0–18·0]). The mean number of modules (stand-alone iCBT 6·9 [SD 6·0]; guided iCBT 9·2 [6·3]) and the mean number of hours worked with the programme (stand-alone iCBT 7·5 [10·2]; guided iCBT 9·8 [11·1]) were higher in the guided iCBT group than in the stand-alone iCBT group.

**Figure 2:**
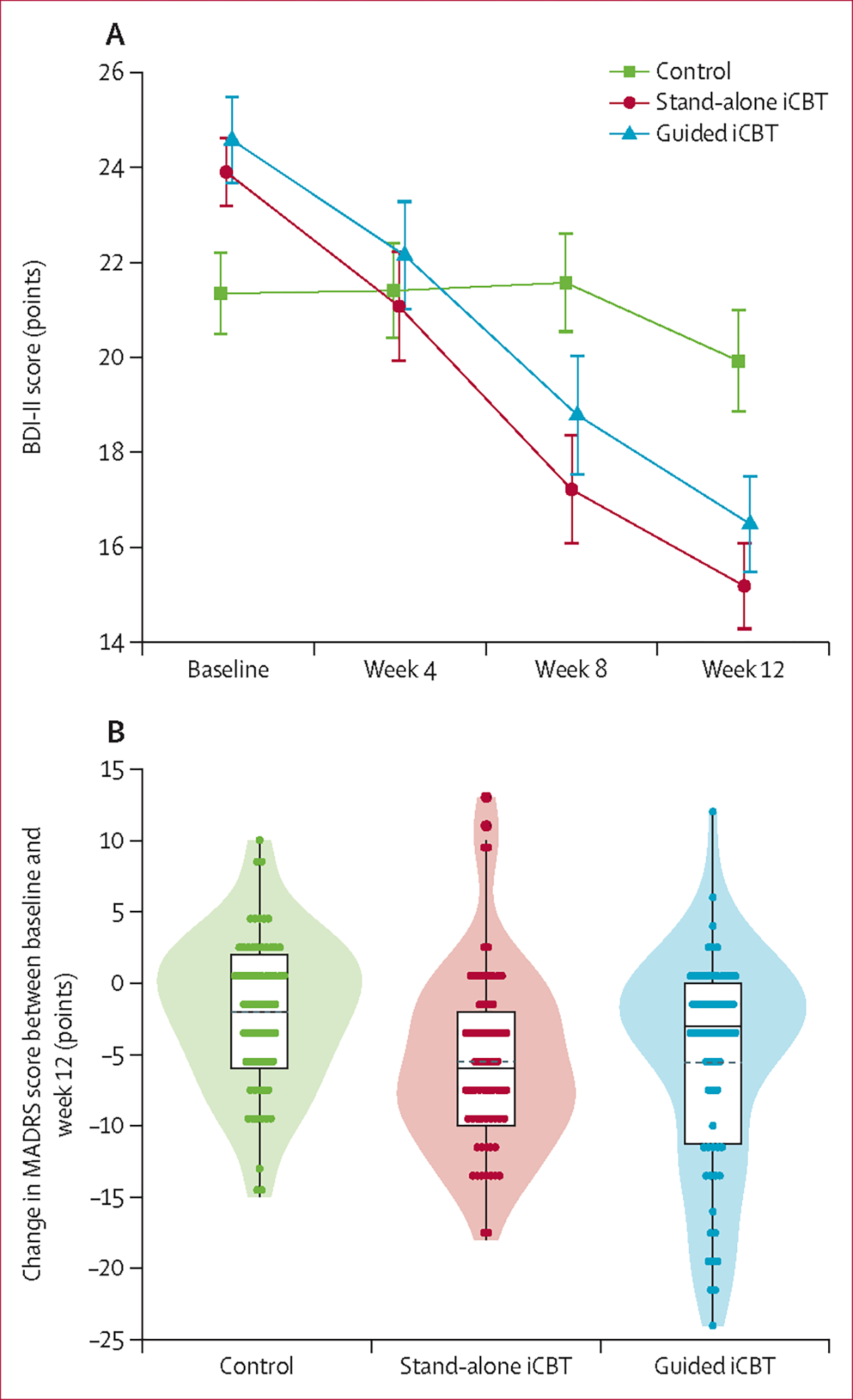
Effects of treatment on the severity of depression symptoms (A) BDI-II score for control and iCBT groups at baseline, week 4, week 8, and week 12. Data are mean (SE). (B) Violin plots showing the change in MADRS score between baseline and end of treatment at week 12. Data shown are IQR (boxes), mean (dotted line), median (solid line), and individual data points (dots). BDI-II=Beck Depression Inventory-II. MADRS=Montgomery-Åsberg Depression Rating Scale.

As shown by the difference in BDI-II score compared with control, significantly reduced depressive symptoms were seen in the stand-alone iCBT (difference 6·32 points [95% CI 3·37–9·27]; p<0·0001; effect size d=0·97 [95% CI 0·64–1·30]) and the guided iCBT (5·80 points [2·71–8·88]; p<0·0001; effect size d=0·96 [0·62–1·30]) groups at week 12 (figure 2, table 3). However, no significant difference was found between the stand-alone iCBT and guided iCBT groups at week 12.

We observed significant improvements in the generic measure of quality of life (WHOQOL-BREF, psychological, physical, and environmental domains) in both iCBT groups compared with the control group (table 4). Similarly, improvements were seen in multiple sclerosis-specific psychological quality-of life assessment (MSIS-29, psychological domain) in both iCBT groups compared with the control group. However, no consistently significant improvements were observed for other domains of generic or multiple sclerosis-specific quality of life in iCBT groups, and no robust effects were seen on measures of fatigue (FSMC and CFS).

We detected no concerns in the predefined safety measures regarding new occurrences of suicidality, as no participant registered a response of 3 on item 9 of the BDI-II at any time point (week 4, week 8, week 12, month 6, or month 12) nor any responses of 3a or 3b on SBQ-R item 3 plus a score of 5 or 6 on SBQ-R item 4 during any of the clinical visits. Accordingly, no concerns for suicidality in the enrolled patients were registered by the study platform during the entire study duration. We also did not detect any hospitalisations due to a psychiatric disorder, suicidality during the clinical interviews, suicidal thoughts mentioned in the message exchange with the study therapists, or any lethal or life-threatening events (including suicide or suicide attempt). Worsening of depressive symptoms during the trial from below to above the cutoff for caseness (BDI-II>13, baseline compared to week 12) was observed in three patients in the control group, one patient in the stand-alone iCBT group, and no patients in the guided iCBT group. Some additional adverse events were recorded during clinical examinations (including pain, urinary retention, diarrhoea, and others), none of which were deemed to be related to the intervention. A detailed list of adverse events is provided in the appendix (p 2).

Treatment effects for the primary endpoint, BDI-II score at week 12, were supported by four sensitivity analyses: ANCOVA with last observation carried forward, ANCOVA with multiple imputations, ANCOVA with complete cases, and linear mixed model for repeated measures using the modified intention-to-treat population. These analyses yielded similar point estimates and effect sizes to the primary analysis (table 3).

Treatment effects on depressive symptoms were substantiated by the clinician-rated MADRS, with significant improvements in both iCBT groups compared with control (table 4, figure 2B). No significant treatment effects were observed on major depressive disorder diagnosis (odds ratio based on logistic regression: iCBT *vs* control 0·62 [95% CI 0·29–13·1], p=0·21; guided iCBT *vs* control 0·62 [0·29–1·32], p=0·22).

Effects on the BDI-II score remained significant at 6 months, as shown by the differences compared with control in stand-alone iCBT (difference 6·79 points [95% CI 2·97–10·60]; p<0·0001; effect size d=0·88 [0·50–1·26]) and guided iCBT (4·99 points [1·04–8·94]; p=0·0030; effect size d=0·68 [0·31–1·05]) groups (appendix p 3), with no significant differences between the two iCBT groups.

The estimated mean difference in BDI-II score after 12 months between non-booster and booster groups was 0·266 points (95% CI −0·851 to 1·380; p=0·64). Both groups exhibited stable BDI-II scores up to month 12 and remained significantly below their baseline levels (all values p<0·0001; appendix p 4).

## Discussion

This trial met its primary endpoint and provides evidence for the efficacy of this multiple sclerosis-specific online depression management tool as a stand-alone or guided application to reduce depressive symptoms in patients with multiple sclerosis over a 12-week period. Both versions were safe and improved domains of quality of life.

Despite the robust treatment effects on depressive symptoms as measured by the BDI-II and the MADRS, we did not observe significant differences in the proportions of participants meeting diagnostic criteria for major depressive disorder. This could in part be due to the fact that our trial was not powered to detect effects in this dichotomous endpoint. Previous work found that affective symptoms (eg, depressed mood and anhedonia) are more likely to remit with treatment, whereas other symptoms (eg, cognitive symptoms) tend to persist.^43^ As such, substantially larger sample sizes might be required to show an effect on the diagnosis of major depressive disorder.

The therapist support provided to participants in the guided iCBT group did not add to the treatment effect in any of the outcome measures investigated. In part, this finding could be attributed to the nature of the therapist support, which was largely geared towards motivation to work with the programme and did not include any actual therapeutic interventions on the therapists’ part. In addition, the proportion of participants receiving psychotherapy at baseline was slightly higher in the guided iCBT group (10%) than in the stand-alone iCBT group (5%), potentially attenuating the effects of iCBT in the guided group. A related question would be if a particular subgroup can be identified in which the therapist support did help substantially (eg, in patients with more severe depression, those with more severe multiple sclerosis, or those who more actively engaged in the exchange with the therapist). Such secondary analyses of our dataset will be conducted in subsequent work.

We were unable to reach the planned sample size of 375 participants (279 were enrolled), at least in part due to the difficulties associated with conducting clinical trials during the COVID-19 pandemic, particularly in a potentially vulnerable group.^44^ Given the robustness of the treatment effect (as supported by several sensitivity analyses of the primary endpoint and similar effects using a clinician-based rating), we are confident that our trial was still sufficiently powered. Further reassurance is provided by the larger effect sizes observed in this phase 3 trial than in our previous single-centre, phase 2 trial.^20^

We observed some differences in the baseline values of the BDI-II scores between the trial groups. Although all statistical analyses accounted for baseline levels of the respective outcome measure, future trials using iCBT in multiple sclerosis could consider using stratified randomisation to minimise the probability of baseline differences. Moreover, average residual BDI-II scores at the end of treatment remained higher than the clinical threshold for depression. This finding indicates that full remission is difficult to reach with an internet-based tool in many cases.

Masking is a key challenge for trials of behavioural interventions, particularly those with patient-reported outcomes. We therefore caution that the observed effects from our study cannot be directly compared with the effects observed in placebo-controlled drug trials for multiple sclerosis-associated depression.^14^ Regardless, results from the patient-reported BDI-II were supported by MADRS scores obtained by masked raters, providing some reassurance that unmasking of participants is unlikely to systematically bias our estimates of treatment effects.

The outcome of a randomised controlled trial depends on the choice of the control condition as much as the experimental treatment.^45^ The literature suggests that a treatment-as-usual or waitlist control condition in behavioural trials is associated with larger effect sizes relative to trials with active comparators.^45^ However, owing to the paucity of evidence for the efficacy of any treatment strategy for multiple sclerosis-associated depression,^13^ we believe that the use of a treatment-as-usual waitlist control group is appropriate.

This trial provides evidence for safety and efficacy of this multiple sclerosis-specific online tool as a stand-alone or guided application to reduce depressive symptoms in multiple sclerosis over a 12-week period. This remote-access, scalable intervention increases the therapeutic options in this patient group and could help to overcome treatment barriers.

## Supplementary Material

Supplementary Appendix

## Figures and Tables

**Table 1:** Baseline characteristics

	Control (n=88)	Stand-alone iCBT (n=95)	Guided iCBT (n=79)

Age	47·3 (11·1)	46·5 (11·9)	47·1 (12·1)
Sex			
Female	69 (78%)	72 (76%)	59 (75%)
Male	19 (22%)	23 (24%)	20 (25%)
Multiple sclerosis disease course			
Relapsing–remitting	65 (74%)	61 (64%)	59 (75%)
Primary progressive	8 (9%)	17 (18%)	9 (11%)
Secondary progressive	14 (16%)	15 (16%)	8 (10%)
PDDS score, points	3 (1–4)	3 (1–4)	3 (2–4)
EDSS score, points	4·0 (2·7–5·3)	4·0 (2·5–5·5)	3·5 (2·5–5)
Time since diagnosis, years	11·1 (7·7)	10·0 (8·1)	11·1 (9·7)
Time since first symptoms, years	13·8 (8·3)	14·3 (9·6)	15·7 (13·0)
Disease-modifying therapies			
Yes	58 (66%)	59 (62%)	48 (61%)
No	30 (34%)	36 (38%)	31 (39%)
Antidepressant treatment			
None	47 (53%)	49 (52%)	48 (61%)
Selective serotonin reuptake inhibitor	25 (28%)	22 (23%)	20 (25%)
Serotonin noradrenaline reuptake inhibitor	10 (11%)	11 (12%)	6 (8%)
Tricyclic antidepressant	2 (2%)	5 (5%)	2 (3%)
Other	7 (8%)	13 (14%)	4 (5%)
Psychotherapy			
Yes	4 (5%)	5 (5%)	8 (10%)
No	84 (95%)	90 (95%)	71 (90%)
BICAMS			
SDMT score, points	50·3 (10·6)	48·7 (12·2)	49·7 (11·5)
CVLT-II score, points	55·3 (10·5)	53·5 (10·9)	52·6 (10·2)
BVMT-R score, points	25·5 (5·7)	25·3 (6·4)	24·5 (7·3)
Ethnicity			
White	61 (69%)	70 (74%)	45 (57%)
African American or Black	0	1 (1%)	4 (5%)
Hispanic or Latino/a	0	1 (1%)	0
Other	0	0	2 (3%)
Not provided	27 (31%)	23 (24%)	28 (35%)

Data are mean (SD), median (IQR), or n (%). BICAMS=Brief International Cognitive Assessment for Multiple Sclerosis. BVMT-R=Brief Visuospatial Memory Test-Revised. CVLT-II=California Verbal Learning Test-II. EDSS=Expanded Disability Status Scale. iCBT=internet-based cognitive behavioural therapy. PDDS=Patient-Determined Disease Steps. SDMT=Symbol Digit Modalities Test.

**Table 2:** Baseline values of outcome measures

	Control (n=88)	Stand-alone iCBT (n=95)	Guided iCBT (n=79)

BDI-II score, points	21·3 (8·2)	23·9 (7·2)	24·6 (8·3)
FSMC score, points	70·6 (17·9)	71·1 (16·7)	72·1 (16·0)
WHOQOL-BREF score, points			
Psychological domain	47·1 (13·6)	42·6 (14·5)	41·7 (14·3)
Physical domain	51·4 (17·7)	49·3 (18·4)	47·4 (18·0)
Social relationships domain	50·9 (21·2)	49·0 (22·3)	50·0 (19·4)
Environment domain	70·0 (14·7)	64·0 (16·4)	65·2 (15·1)
CFS score, points	21·1 (5·3)	21·6 (5·9)	21·5 (5·4)
MADRS score, points	17·7 (6·5)	19·6 (6·1)	20·5 (7·8)
MSIS psychological domain score, points	47·3 (19·0)	54·2 (16·2)	53·4 (19·1)
Diagnosis of major depressive disorder			
Yes	51 (58%)	60 (63%)	59 (75%)
No	37 (42%)	35 (37%)	20 (25%)

Data are mean (SD) or n (%). BDI-II=Beck Depression Inventory-II. CFS=Chalder Fatigue Scale. FSMC=Fatigue Scale for Motor and Cognitive Functions.

MADRS=Montgomery-Åsberg Depression Rating Scale. MSIS=Multiple Sclerosis Impact Scale. WHOQOL-BREF=WHO Quality of Life-Brief Version.

**Table 3:** Effects of treatment on depressive symptoms at week 12 (primary endpoint)

	Estimate (95% CI)	p value	Cohen’s d (95% CI)

**Linear mixed model for repeated measures (ITT population)** *			
Control *vs* stand-alone iCBT	6·32 (3·37–9·27)	<0·0001	0·97 (0·64–1·30)
Control *vs* guided iCBT	5·80 (2·71–8·88)	<0·0001	0·96 (0·62–1·30)
**ANCOVA with last observation carried forward (ITT population)** †			
Control *vs* stand-alone iCBT	5·81 (3·38–8·24)	<0·0001	0·90 (0·59–1·20)
Control *vs* guided iCBT	5·58 (3·02–8·13)	<0·0001	0·89 (0·57–1·21)
**ANCOVA with multiple imputations (ITT population)** †			
Control *vs* stand-alone iCBT	6·32 (4·20–8·45)	<0·0001	0·99 (0·67–1·31)
Control *vs* guided iCBT	5·71 (3·48–7·94)	<0·0001	0·94 (0·60–1·27)
**ANCOVA with complete cases** †			
Control *vs* stand-alone iCBT	6·36 (3·85–8·86)	<0·0001	0·99 (0·66–1·33)
Control *vs* guided iCBT	5·93 (3·33–8·54)	<0·0001	0·97 (0·63–1·32)
**Linear mixed model for repeated measures (mITT population)** †			
Control *vs* stand-alone iCBT	6·41 (3·37–9·46)	<0·0001	0·95 (0·61–1·30)
Control *vs* guided iCBT	5·97 (2·88–9·07)	<0·0001	0·94 (0·60–1·29)

Data are the difference in BDI-II score (points) between the two groups specified at week 12. BDI-II=Beck Depression Inventory-II. iCBT=internet-based cognitive behavioural therapy. ITT=intention to treat. mITT=modified intention to treat.

* Prespecified primary analysis.

† Prespecified sensitivity analysis.

**Table 4:** Secondary and exploratory endpoints

	Estimate (95% CI)	p value	Cohen’s d (95% CI)

**WHOQOL-BREF psychological domain**			
Control *vs* stand-alone iCBT	−8·29 (−12·50 to −4·08)	<0·0001	−0·78 (−1·10 to −0·45)
Control *vs* guided iCBT	−7·95 (−12·32 to −3·59)	<0·0001	−0·76 (−1·10 to −0·42)
**WHOQOL-BREF physical domain**			
Control *vs* stand-alone iCBT	−6·99 (−11·18 to −2·82)	0·0002	−0·62 (−0·94 to −0·29)
Control *vs* guided iCBT	−6·05 (−10·37 to −1·72)	0·0021	−0·59 (−0·92 to −0·25)
**WHOQOL-BREF social relationships domain**			
Control *vs* stand-alone iCBT	−5·31 (−10·26 to −0·37)	0·0164	−0·41 (−0·73 to −0·09)
Control *vs* guided iCBT	−2·32 (−7·41 to 2·77)	0·25	−0·19 (−0·52 to 0·13)
**WHOQOL-BREF environmental domain**			
Control *vs* stand-alone iCBT	−4·46 (−7·84 to −1·08)	0·0035	−0·57 (−0·89 to −0·25)
Control *vs* guided iCBT	−4·08 (−7·55 to −0·61)	0·0091	−0·52 (−0·85 to −0·19)
**MSIS psychological domain**			
Control *vs* stand-alone iCBT	9·02 (3·61 to 14·43)	0·0003	0·76 (0·43 to 1·09)
Control *vs* guided iCBT	7·77 (2·21 to 13·33)	0·0021	0·62 (0·29 to 0·95)
**MSIS physical domain**			
Control *vs* stand-alone iCBT	3·70 (0·15 to 7·24)	0·0197	0·39 (0·07 to 0·71)
Control *vs* guided iCBT	2·57 (1·09 to 6·22)	0·10	0·30 (−0·03 to 0·63)
**FSMC**			
Control *vs* stand-alone iCBT	2·93 (−0·57 to 6·42)	0·0574	0·30 (−0·02 to 0·62)
Control *vs* guided iCBT	1·94 (−1·66 to 5·54)	0·19	0·22 (−0·11 to 0·55)
**CFS**			
Control *vs* stand-alone iCBT	1·49 (−0·23 to 3·02)	0·0495	0·29 (−0·03 to 0·61)
Control *vs* guided iCBT	0·20 (−1·56 to 1·97)	0·56	0·03 (−0·30 to 0·35)
**MADRS**			
Control *vs* stand-alone iCBT	3·16 (0·84 to 5·48)	0·0026	0·59 (0·25 to 0·94)
Control *vs* guided iCBT	2·98 (0·61 to 5·35)	0·0053	0·54 (0·19 to 0·90)

Data are the difference in score (points) between the two groups specified on the stated scales at week 12. CFS=Chalder Fatigue Scale. FSMC=Fatigue Scale for Motor and Cognitive Functions. iCBT=internet-based cognitive behavioural therapy. MADRS=Montgomery-Åsberg Depression Rating Scale. MSIS=Multiple Sclerosis Impact Scale. WHOQOL-BREF=WHO Quality of Life-Brief Version.

## Data Availability

Anonymised individual patient data from the primary trial phase—including data on treatment group, region (Germany or the USA), and measurements of primary and secondary endpoints—are available on request at https://zenodo.org/record/7965979.

## Research in context

### Evidence before this study

We searched PubMed, PsycINFO, the Cochrane library, and Embase from database inception to March, 2018, using keywords “Multiple Sclerosis”, “psychological distress”, “stress reduction”, “distress”, “depressive symptoms”, and “anxiety symptoms”, yielding 15 trials of cognitive behavioural therapy (CBT) or mindfulness-based therapy for the treatment of depressive symptoms in multiple sclerosis. The analysis indicated that behavioural interventions such as CBT can be effective to reduce depression when delivered in person or remotely with moderate effect size, compared with treatment-as-usual, waitlist, or active-treatment control conditions. However, the overall quality of the trials was low, sample sizes were small, and considerable heterogeneity was present. We previously conducted one single-centre trial in Germany of internet-based CBT (iCBT) for reducing depressive symptoms in multiple sclerosis that showed good safety and efficacy. These findings support the potential of iCBT for the treatment of depression associated with multiple sclerosis but also highlight the need for large confirmatory multicentre trials that could inform clinical practice.

### Added value of this study

We conducted a multicentre randomised controlled trial in two countries to test the efficacy of a multiple sclerosis-specific online depression-management tool. Compared with treatment as usual, we found a significant reduction in depressive symptoms after 12 weeks of treatment with either a stand-alone or a guided version of the tool, added to treatment as usual. The trial also showed beneficial effects on generic as well as multiple sclerosis-specific quality-of-life measures. Treatment effects were maintained at 6 months and at 12 months. No safety signals for increased risk of suicidality or other adverse events were detected.

### Implications of all the available evidence

The available evidence supports the use of CBT-based treatments for depression associated with multiple sclerosis and suggests that scalable, fully automated CBT programmes, delivered using the internet, are effective in reducing depressive symptoms in these patients. Such programmes could be particularly helpful to facilitate access for patients who are unable to receive CBT in person.
