# Supplementary material for: Internet-delivered cognitive behavioural therapy programme to reduce depressive symptoms in patients with multiple sclerosis: a multicentre, randomised, controlled, phase 3 trial
Source: Lancet Digit Health. Author manuscript; Available in PMC 2024 Mar 8. (PMC10921847; doi:10.1016/S2589-7500(23)00109-7)
Supplement: Supplementary Appendix [file NIHMS1966844-supplement-Supplementary_Appendix.pdf]

THE LANCET
Digital Health

Supplementary appendix

This appendix formed part of the original submission and has been peer reviewed. We post it as supplied by the authors.

Supplement to: Gold SM, Friede T, Meyer B, et al. Internet-delivered cognitive behavioural therapy programme to reduce depressive symptoms in patients with multiple sclerosis: a multicentre, randomised, controlled, phase 3 trial. *Lancet Digit Health* 2023; **5:** e668–78.

Table of contents

Supplementary Table 1

Supplementary Figures 1 and 2

Supplementary Materials Program Development & Piloting

Therapist Mannual

Statistical Analysis Plan

Trial Protocol

Supplementary Table 1: Adverse events

	Control	iCBT	guided iCBT
**New occurrence of suicidality**	0	0	0
**Worsening of depressive symptoms**	3	1	0
Other (see below for details)	6	5	3
Back pain in the context of M. Bechterew, shoulder pain, osteopenie, spondylarthritis (exarcerbation)	*1*	*0*	*0*
Urinary retention	*1*	*0*	*0*
Coughing; subcutanous mastectomie / liposection due to gynecomastia	*1*	*0*	*0*
Persistent menstrual bleeding	*0*	*0*	*1*
Face phlegmon, abscess (MRSA pos,)	*1*	*0*	*0*
Frequent diarrhea, urinary incontinence	*0*	*1*	*0*
Diarrhea	*0*	*1*	*0*
Pain and swelling in lower back near buttock (grade moderate) tx = surgical incision + drainage of pustule	*1*	*0*	*0*
Lower back pain (grade moderate), no tx	*0*	*1*	*0*
Fall on stairs leading to left tibial plateau fracture	*1*	*0*	*0*
Lower back pain radiating into left leg, started physical therapy	*0*	*0*	*1*
Urinary urgency + urinary tract infection (grade mild).	*0*	*1*	*0*
Chest pain, globus sensation	*0*	*1*	*0*
Abdominal pain with history of endomertriosis partial hysterectomy	*0*	*0*	*1*

Supplementary Figure 1: Effects on depressive symptoms (BDI-II) at 6-months follow-up (ITT population).

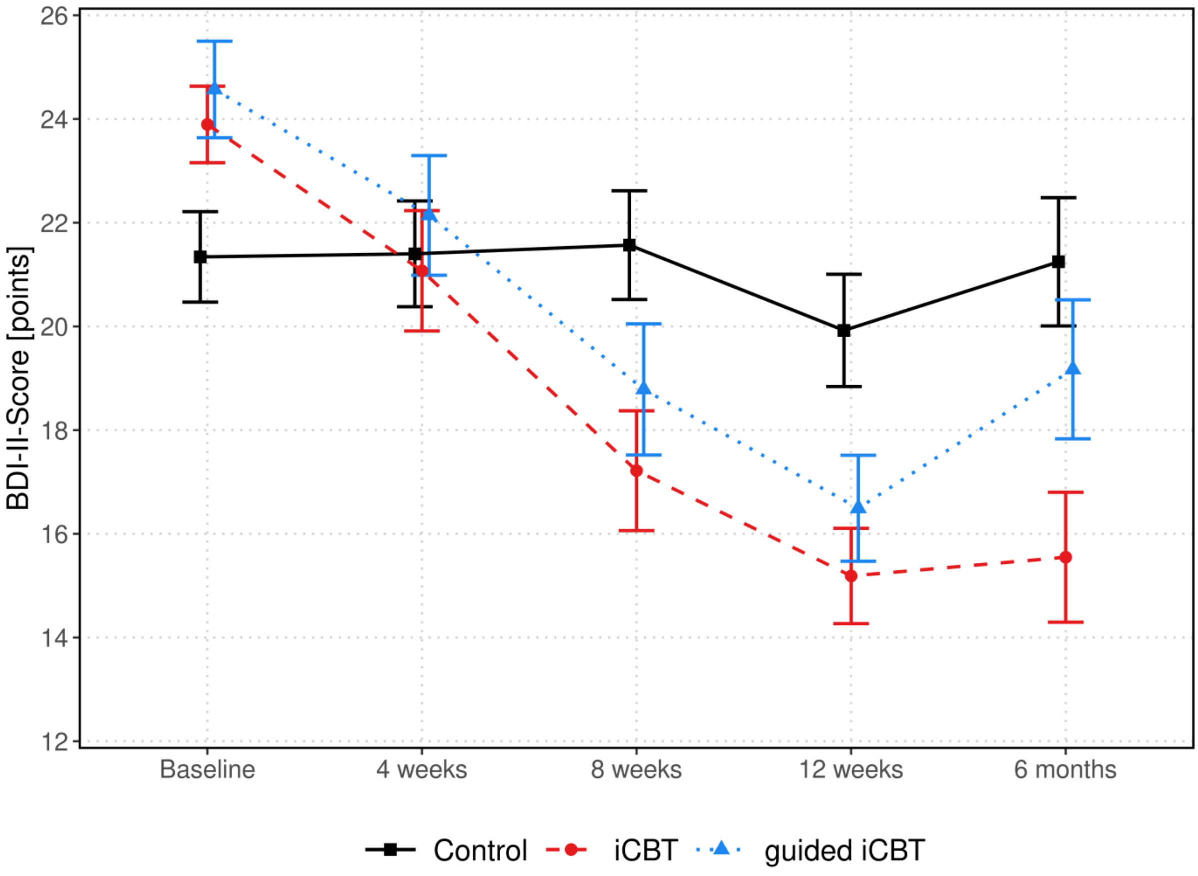

Supplementary Figure 2: Long-term outcome of depressive symptoms (BDI-II) up to 12-months follow-up shown for patients who completed either version of iCBT (guided or stand alone) during the primary trial phas (baseline to month 3), shown separately for those receiving booster sessions vs. no booster sessions during the extension/maintenance phase of the trial.

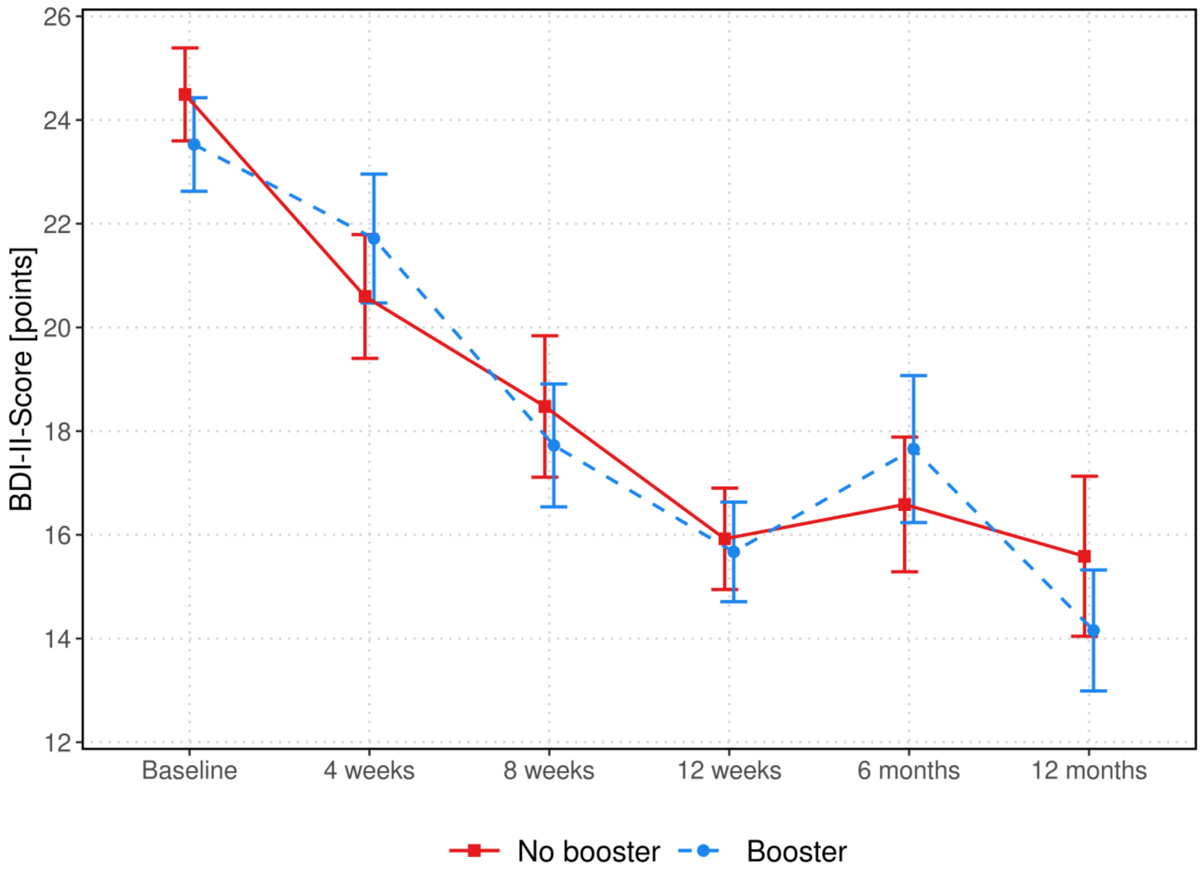

Description of program development, pilot evaluation and therapist support manual

a. Development of the online program

A previous randomized controlled trial with the digital depression treatment program "deprexis" had shown promising results[1]. In that two-arm trial (deprexis vs. waitlist control group) with *n* = 90 patients with MS, a significant treatment effect on depression reduction (BDI) over 9 weeks had been demonstrated, with a moderate between-groups effect size of *d* = 0.53. However, despite these encouraging results on depressive symptom reduction, in a survey of n=34 of the participants in this trial, more than half of the trial participants (n=18, 51%) had suggested that the program might be improved by developing content adjustments to better suit the needs of patients with MS.

Therefore, we developed and pilot-tested a modified MS-specific digital depression treatment program, which was based on deprexis, but which also differs substantially because it contains MS-specific content. Additional content and functional modifications were also required to improve the content coherence and user navigation, given that the new program specifically targets depression in MS, rather than depression more generally. To differentiate this new program from deprexis, and to avoid potential confusion between these different digital interventions, a new name—*amiria*—has now been given to the new, MS-specific intervention.

The major content modifications made to *amiria* can be summarized as follows:

(1) Unlike deprexis, which targets depression in the general population, amiria clarifies in the first module that it is intended specifically for MS patients;

[1] Fischer, A., Schröder, J., Vettorazzi, E., Wolf, O. T., Pöttgen, J., Lau, S., ... & Gold, S. M. (2015). An online programme to reduce depression in patients with multiple sclerosis: a randomised controlled trial. *The Lancet Psychiatry, 2(3),* 217-223.

(2) several MS illness parameters are queried early on in an interactive amiria sequence, including time since diagnosis, symptom severity, and subjective impairment;

(3) an interactive sequence introduces the concept that biological as well as psychosocial factors might contribute to depression in MS;

(4) a psychoeducational sequence introducing a cognitive-behavioral model of depression has been introduced. In this sequence, users can reflect, for example on whether they tend to have more optimistic or pessimistic cognitive responses to having MS (e.g., "Having MS makes me appreciate every day even more" vs. "Having MS means the future is bleak and hopeless for me");

(5) a section on subjective reasons for depression has been revised, such that having MS is included as a potential reason for depression onset;

(6) a section in which previous research is discussed has been developed and mentions the previous trial conducted with persons with MS (Fischer et al., 2015).

In addition to these major changes, content changes were also made to all other program modules. These included the addition of MS-related information, illustrative case example, relevant response options, and program reactions to new response options. Examples of these modifications include:

- The module on behavioral activation and activity scheduling acknowledges that certain activities may be inappropriate because of the MS diagnosis, and users are encouraged to select only activities that they feel are safe.
- In the module on cognitive modification, several examples of MS-specific automatic thoughts were added. This includes, for example, the following text passage:

    - *"Thoughts can trigger feelings: If I take my medication and then think, 'This means that something is wrong with me', I might feel sad. If I think, 'Taking this medication helps me stay well', then I might feel more hopeful.*
- In the module on acceptance and mindfulness techniques, MS-related case examples were added to illustrate certain therapeutic principles, such as identifying and pursuing personally important values. For example, the following text passage was added:
    - *"Sarah has relapsing remitting MS. When she was first diagnosed with MS she put her life on hold. She wanted to make sure she was prepared for managing future relapses. While Sarah felt that she was managing her physical health well she realized that emotionally something was missing. She was no longer following her creative passions. This helped Sarah to get some perspective on what was important to her. She began attending art classes once a week and reconnected with her passion for art and friendships."*
- In the module on interpersonal relationships, MS-related response options as well as program reactions were added. For example:
    - New response option: *"Now that I have MS, I avoid my friends..."*
    - New program reaction to this response: *"I completely understandable that you feel like withdrawing from others due to your MS. However, avoiding others can be like a vicious cycle that leads into social isolation and feeling even worse. Let's see if we can think of alternative ways, okay?"*
- In a section of the "positive psychology module" that discusses personal strengths and talents, MS-related content was added. For example:

- *"Having MS and facing the difficulties and challenges associated with this disease takes a lot of courage! Doing this program is a sure sign that you do have this kind of courage and are facing this issue, which is great to see!"*

Booster Sessions

An additional modification made to the program concerned the development of three "booster sessions," which were designed to provide additional CBT content that is compatible with but goes beyond content already covered in previous program modules. Specifically, the following content is addressed in these three booster modules:

1. Cognitive modification: The first booster session begins with a brief discussion of the "ABC" model, according to which activating situations or events (A) can trigger certain automatic thoughts or beliefs (B) that then lead to certain emotional consequences (C). Examples relevant to MS are discussed to illustrate the idea that thoughts or beliefs play a critically important role in producing different kinds of emotional consequences (e.g., a thought like "If my body is not performing well, the day is ruined and things will get worse" may tend to trigger unpleasant emotions). Techniques are discussed to help patients gain a sense of mental distance from unhelpful automatic thoughts (e.g., "Hold that thought in your mind for just a few seconds... And now try to imagine you're literally holding that thought in your hand, just looking at it..."). The module also introduces the idea that unhelpful automatic thoughts could be systematically distorted, and might fit one or several categories of common "cognitive distortions" (e.g., "fortune telling, all-or-nothing thinking, discounting the positive, counterfactual thinking"). Techniques to help program users challenge or refute unhelpful thoughts or to distract themselves from such thoughts are discussed (e.g., taking the "bird's eye view" to gain mental distance, engaging in physical exercise or activities to distract oneself from ruminative

thoughts). This first booster session also includes a worksheet to help users practice the cognitive modification techniques discussed.

2. Behavioral techniques (healthy lifestyle habits): The second booster session reiterates the idea that engaging in certain actions of behaviors could be useful in preventing or reducing depressive symptoms. The session focuses on lifestyle-related behavioral habits, including healthy nutrition, regular exercise, sleep hygiene and engaging in potentially pleasurable activities. Fictional case examples or discussed to illustrate how unhealthy nutrition and a sedentary lifestyle can contribute to depression. Principles of generally healthy eating are interactively explored (e.g., avoiding too many industrially processed foods with excessive amounts of added sugars, trans-fatty acids, etc.). Benefits of physical activity and general recommendations for physical activity are also discussed and interactively explored. Program users are encouraged to set personally relevant, achievable and specific goals (e.g., "Monday, 6 pm: Go on a walk in nature for 30-45 minute) and to review potential obstacles and strategies for overcoming them ("mental contrasting"). Principles of sleep hygiene, such as committing to regular sleep and wake-up times and avoiding excessive daytime napping are also discussed. Furthermore, a list of potentially pleasurable activity suggestions is provided, and users are encouraged to schedule sufficient time for them in their daily lives.
3. Mindfulness meditation and cognitive defusion: The third booster module focuses on the topic of mindfulness and cognitive defusion. The module begins with a breathing exercise in which users are encouraged to merely observe thoughts and feelings from a detached perspective. Exercises inspired by ACT (acceptance and commitment therapy) are introduced, as these are also compatible with content covered in previous modules (e.g., the exercise known as "soldiers on a parade" in which users imagine that toy soldiers are parading in front of their mind's eye,

"holding a flag with whatever the thought says"). Potential benefits of regular mindfulness meditation practice are discussed, and information is offered for users who wish to learn more about this topic. The module closes with a brief review of the content covered in all three "booster sessions."

The program changes were developed by an experienced licensed clinical psychologist with training in cognitive-behavioral therapy (B.M.) and experience in the development of a CBT-based internet intervention targeting MS-fatigue ("Elevida"). Feedback on the modifications was provided by a clinical psychologist with extensive experience in treating psychological aspects of MS (R.M.-M.), a psychologist with training in behavior therapy and experience in treating persons with MS (J.H.) and a neurologist (C.H.). The modifications made to the program were also discussed intensively in several e-mail exchanges and personal meetings, in which primarily B.M. and J.H. participated. Program modifications were reviewed and further revised during an in-person meeting at King's College, London.

Of note, a broad range of relevant material was reviewed and informed the development of the program modifications[2], and no content was taken from any one previously existing

---

[2] Relevant literature that was reviewed in this process included, among others, the following articles:

Arnett, P. A., Barwick, F. H., & Beeney, J. E. (2008). Depression in multiple sclerosis: review and theoretical proposal. Journal of the International Neuropsychological Society, 14(5), 691-724.
Cosio, D., Jin, L., Siddique, J., & Mohr, D. C. (2011). The effect of telephone-administered cognitive–behavioral therapy on quality of life among patients with multiple sclerosis. Annals of Behavioral Medicine, 41(2), 227-234.
Dennison, L., & Moss-Morris, R. (2010). Cognitive–behavioral therapy: what benefits can it offer people with multiple sclerosis?. Expert review of neurotherapeutics, 10(9), 1383-1390.
Graziano, F., Calandri, E., Borghi, M., & Bonino, S. (2014). The effects of a group-based cognitive behavioral therapy on people with multiple sclerosis: a randomized controlled trial. Clinical rehabilitation, 28(3), 264-274.
Hind, D., Cotter, J., Thake, A., Bradburn, M., Cooper, C., Isaac, C., & House, A. (2014). Cognitive behavioural therapy for the treatment of depression in people with multiple sclerosis: a systematic review and meta-analysis. BMC psychiatry, 14(1), 1-13.
Feinstein, A. (2011). Multiple sclerosis and depression. Multiple Sclerosis Journal, 17(11), 1276-1281.
Feinstein, A., Magalhaes, S., Richard, J. F., Audet, B., & Moore, C. (2014). The link between multiple sclerosis and depression. Nature Reviews Neurology, 10(9), 507-517.
Heesen, C., Köpke, S., Kasper, J., Poettgen, J., Tallner, A., Mohr, D. C., & Gold, S. M. (2012). Behavioral interventions in multiple sclerosis: a biopsychosocial perspective. Expert review of neurotherapeutics, 12(9), 1089-1100.
Hind, D., O'Cathain, A., Cooper, C. L., Parry, G. D., Isaac, C. L., Rose, A., ... & Sharrack, B. (2010). The acceptability of computerised cognitive behavioural therapy for the treatment of depression in people with chronic physical disease: a qualitative study of people with multiple sclerosis. Psychology and Health, 25(6), 699-712.
Mohr, D. C., Likosky, W., Bertagnolli, A., Goodkin, D. E., Van Der Wende, J., Dwyer, P., & Dick, L. P. (2000). Telephone-administered cognitive–behavioral therapy for the treatment of depressive symptoms in multiple sclerosis. Journal of consulting and clinical psychology, 68(2), 356.
Moss-Morris, R., Dennison, L., & Chalder, T. (2010). Supportive adjustment for multiple sclerosis (SaMS). London: MS Society-funded research, university of Southampton and kings college.
Moss-Morris, R., Dennison, L., Landau, S., Yardley, L., Silber, E., & Chalder, T. (2013). A randomized controlled trial of cognitive behavioral therapy (CBT) for adjusting to multiple sclerosis (the saMS trial): does CBT work and for whom does it work?. Journal of consulting and clinical psychology, 81(2), 251.

source. The collaborating parties had agreed in writing that intellectual property of all program content, including the modifications to improve the suitability of the program for patients with Multiple Sclerosis, would remain with GAIA, the developer, owner, and operator of the program evaluated in this trial.

The program explicitly acknowledged the contribution of the collaborating partners in the following text, which could be accessed via the program menu: *"Gaia would like to thank the psychologists, physicians and patients who provided valuable input for the development of this program. Specifically, we would like to acknowledge and thank Professor Rona Moss-Morris and Dr Joanna Hudson at Institute of Psychiatry, King's College London, for their detailed and constructive review and suggestions."*

Pakenham, K. I. (2008). Making sense of illness or disability: The nature of sense making in multiple sclerosis (MS). Journal of Health Psychology, 13(1), 93-105.
Siegert, R. J., & Abernethy, D. (2005). Depression in multiple sclerosis: a review. Journal of Neurology, Neurosurgery & Psychiatry, 76(4), 469-475.
Vaughan, R., Morrison, L., & Miller, E. (2003). The illness representations of multiple sclerosis and their relations to outcome. British journal of health psychology, 8(3), 287-301.
Wallin, M. T., Wilken, J. A., Turner, A. P., Williams, R. M., & Kane, R. (2006). Depression and multiple sclerosis: Review of a lethal combination. Journal of Rehabilitation Research & Development, 43(1).

b. Pilot Evaluation of the program

A pilot evaluation of the modified program version was conducted at the University Medical Center Hamburg-Eppendorf with *n*=8 MS-patients, 7 of whom had participated in a previous study on this intervention. Results were encouraging; on average, 78% endorsed positive evaluative statements, such as “the content seemed correct, interesting, and helpful” (see Figure 1). Of note, all 8 patients agreed that “this program could be helpful for people living with MS” and that it was overall “good and interesting”.

Respondents were also asked to elaborate on their subjective impressions of the modified program version (qualitative impressions). Responses were generally consistent with the quantitative findings and confirmed the impression that the patients were satisfied with the modified program version. All responses (translated but unedited) are provided in Table 1.

Figure 1: Quantitative results of the pilot evaluation of the modified program version with *N*=8 MS patients.

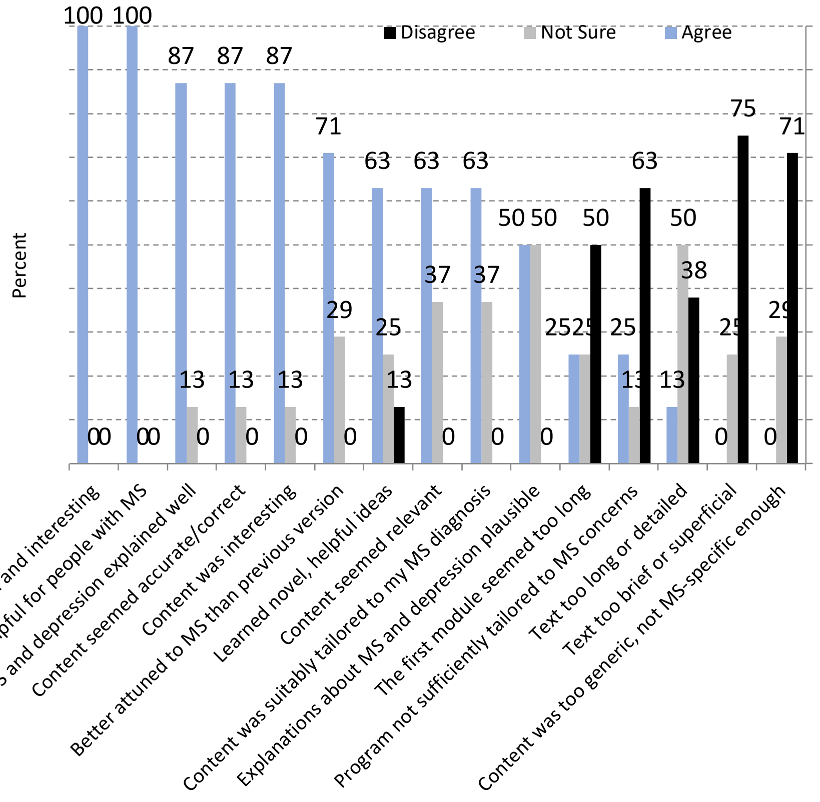

Table 1. Quantitative results of the pilot evaluation of MS-Deprexis with N=8 MS patients.

**Question:** Could you describe your impressions of the revised program? What did you like and what could still be improved? Any ideas or suggestions are welcome!	
1.	Compared to the first version, this is a significant improvement, and it seems more professional. The current version clearly discusses MS in more detail; it's not just dismissed as something that may or may not play a role in depression. It is also acknowledged that depression may be associated with physical limitations; this was not the case previously.
2.	The revised program is much clearer. Personally, I find that it's good and sufficiently tailored to meet the needs of people with MS. It seems quite personal, and one can better understand how the individual sequences are linked with each other. The way it's structured now, I think one can work very well with it. I found the suggestions to overcome one's sense of lethargy quite supportive. I can't think of any suggestions for further improvements. I hope I can soon use this program without any time limitations. I was happy to work with it again.
3.	It's quite okay the way it is now. I suggest that you focus even more on symptoms and what to do if you experience any.
4.	I found this version more appealing than the first (as far as I remember).
5.	To answer this question, I would have to use the program over a longer period.
6.	I think the meditation suggestions should be spoken [as an audio recording], as this would make it easier to calm down. Other than that, the text and the questions seemed pretty transparent and manipulative, but when you open yourself up to it, it's alright. After all, it's just a computer program.

7. Good: 1. Exercise with the movie theater ("thoughts on the screen") Topic: gaining distance from thoughts, A) Distance to feelings (goal: observing our own thoughts as they pass by on the screen, relaxation, thoughts and feelings - observe without effort) B) thoughts and emotions still float by on the movie screen (goal: increasing tranquility / being a mindful observer, looking at thoughts and feelings, just perceiving without judgment, goal: gaining distance from the situation, adopting a bird's eye view) 2. Relaxation exercise (inhaling and exhaling). The exercises are a welcome part of the first module, after quite a bit of text. The introductions to the topics are interesting and lightened up by the pictures, but some passages seem too lengthy. It would be hard to make it through the first module in 30 minutes. I think at least one hour should be scheduled for the first chat. I tested the program on a desktop computer - like the first time. Compared to the first version of the program, I found no differences in the design of the pages. The pages are bright and illustrated with pictures that break up the text. I also did the mood check, the mood check questionnaire covering the past 2 weeks (PHQ-9), which is the depression check.

8. As a young MS patient I found the program very appealing. There are many interesting and professional facts in it, but it's not described in excessive detail. I liked very much that you can select different responses. The program is very simple and easy to understand. What made it a bit difficult for me is that I currently do not have depression. Since many questions really focus on depression, this may appeal more to people with acute depression. This made me feel a bit like I wasn't using the right program for me. Having MS makes me feel very sad and thoughtful some days, but I'm not depressed. I am pleased, nevertheless, that you're doing so much for MS research.

c. Description of therapist manual

A "therapist support manual" was developed specifically for this trial. This manual was developed and is owned by GAIA and revised in collaboration with the psychologists involved in providing support and supervision in the respective arm of the trial. Prior to the trial start, a one-day training workshop was held at King's College, London, in which the three therapists and two supervisors involved in the provision of patient support participated.

To convey an impression of the manual, an abbreviated version is reproduced on the following pages. References to specific names as well as technical procedures specific to the program or the trial have been deleted from this abbreviated version.

IDEMS trial:

Therapist Support Manual

(ABBREVIATED VERSION)

Table to Contents

Introduction 12

Basic principles for providing web-message support in the IDEMS trial 13

- Accessing the secure online study platform 13
- Technical problems or questions 13

Basic principles of therapeutic web-messaging 13

- Positive framing: Focus on strengths and resources rather than only problems 13
- Relevant literature for further reading 13

General structure of messages: Standardized plus individualized elements 15

The standardized message elements 15

Weekly messages - Example „Welcome message“ 15

Writing weekly feedback messages 18

Example of Complete Message 20

MS-specific feedback and responses to queries 20

Introduction

This manual is intended for "therapists" participating in the IDEMS trial (https://clinicaltrials.gov/ct2/show/NCT02740361). In the context of this study, the term "therapists" refers to study personnel involved in providing web message support to participants in the respective condition of the trial. The main tasks of these therapists will be to use the online study platform to (1) send weekly messages to participants and (2) respond within three days to any web-messages they receive from participants.

This manual was based, in part, on a previous manual developed by Thomas Berger in the context of another study in which e-mail support was provided for Deprexis. As described in the IDEMS study protocol, the e-mail support will generally follow procedures used by Berger et al. (2011).

Basic principles for providing web-message support in the IDEMS trial

- **Accessing the secure online study platform**
  The secure online study platform, through which all web-messages are sent and received, can be accessed at *[web address deleted here because it is no longer accessible]*.

- **Technical problems or questions**
  If you experience any technical problems with the platform or notice that something doesn't work, please e-mail *[contact persons]*.

Basic principles of therapeutic web-messaging

Behaviours used by therapists in the context of supporting internet-based CBT interventions were described by Paxling et al. (2013, see Table 1). Based on this analysis, therapists can:

- reassure clients that tasks can be completed flexibly,
- praise them for their effort or progress,
- express interest in the client's life and activities,
- remind them of tasks or program elements,
- educate them about symptom-related issues,
- self-disclose relevant information,
- convey the positive expectation that practicing will likely lead to improvement,
- and they can empathically express that they understand the clients' situation

Two of these behaviours correlated with better outcome in Paxling's study: deadline flexibility and task reinforcement. Four behaviours correlated with engagement (module completion): task reinforcement, task prompting, self-efficacy shaping, and empathetic utterance. Therefore, it seems advisable that therapists can use these behaviours flexibly and appropriately. However, in the context of this study, therapists should refrain from providing extensive advice, giving detailed explanations or getting involved in lengthy discussions about specific problem situations. The purpose of the messages is to support and guide patients' independent use of the internet intervention – not to provide a therapeutic intervention in itself.

Source: Paxling, B., Lundgren, S., Norman, A., Almlöv, J., Carlbring, P., Cuijpers, P., & Andersson, G. (2013). Therapist behaviours in internet-delivered cognitive behaviour therapy: analyses of e-mail correspondence in the treatment of generalized anxiety disorder. Behavioural and Cognitive Psychotherapy, 41(03), 280-289.

- **Positive framing: Focus on strengths and resources rather than only problems**
  Another basic principle, based on previous work by Thomas Berger, is that the web messages should aim to motivate participants and emphasize positive aspects, such as strengths, accomplishments and individual resources. The messages should generally highlight positive aspects (such as participants' work with the program, their goals and intentions, their abilities, their interpersonal talents or resources, successful mastery of difficult life circumstances, etc.). If problems are discussed at all, they should always be embedded in a context of "resource activating" (or positively framed) interventions.

- **Relevant literature for further reading**

A few studies have analyzed how e-mail support can or should be provided in the context of internet interventions for depression or chronic health conditions. These papers may be worth reading:

Svartvatten, N., Segerlund, M., Dennhag, I., Andersson, G., & Carlbring, P. (2015). A content analysis of client e-mails in guided internet-based cognitive behavior therapy for depression. Internet Interventions, 2(2), 121-127.

This was an interesting content analysis of e-mails sent by patients to therapists in a Swedish internet treatment study. They found that certain types of patient statements correlated fairly strongly with intervention usage and symptomatic improvement. Specifically, statements conveying that the patient regarded an exercise as plausible and likely useful (they called this "alliance") predicted engagement and pre-post benefit. Thus, if a patient writes: "The suggestions in this module really made sense to me. If I regularly engage in activities that satisfy my need for self-esteem and competence, then I will probably feel much better!" – this could be taken as a positive prognostic sign. A second category that correlated with engagement and positive outcome was "observing positive consequences". Thus, if a patient writes things like: "I tried the exercise, and it really did help!", then this is a very good prognostic sign. In other words, these are the kinds of statements that are ideal! Other kinds of statements, such as "I didn't understand this" or "I didn't get what (or why) I was supposed to do…" or "I don't think this will help" probably indicate problems and a worse prognosis.

Dirkse, D., Hadjistavropoulos, H. D., Hesser, H., & Barak, A. (2015). Linguistic analysis of communication in therapist-assisted internet-delivered cognitive behavior therapy for generalized anxiety disorder. Cognitive behaviour therapy, 44(1), 21-32.

This linguistic analysis of iCBT for generalized anxiety disorder suggested that certain words tend to reduce over time in patients' written communication – such as negative emotion words. However, "past tense" words actually increased. This might suggest that as patients' symptoms diminish and positive moods return, their language will reflect this, and they may start to write about their problems or "old self" as something that is now in the past, no longer current (e.g., "When I was still struggling with depression, I used to sit at home all day and think about how pointless everything is, whereas now I make sure to get out and see my friends, which is much more helpful!").

Pugh, N. E., Hadjistavropoulos, H. D., Klein, B., & Austin, D. W. (2014). A case study illustrating therapist-assisted internet cognitive behavior therapy for depression. Cognitive and Behavioral Practice, 21(1), 64-77.

An interesting case study in which the authors go through some very detailed examples of how different modules in an internet treatment can be discussed by an e-mail therapist who supports the program. This could be quite instructive as many of the modules in this treatment resemble those included in deprexis.

Holländare, F., Gustafsson, S. A., Berglind, M., Grape, F., Carlbring, P., Andersson, G., ... & Tillfors, M. (2016). Therapist behaviours in internet-based cognitive behaviour therapy (ICBT) for depressive symptoms. Internet Interventions, 3, 1-7.

Another content analysis of therapist behaviors in guided internet treatment. Two categories were found to be particularly useful: (1) Affirming ("You're right…", "What you experience is quite common for people who…"), (2) Encouraging ("Great to hear that you did this…" or "It sounds great that you went to the movies this past weekend…").

Schneider, L. H., Hadjistavropoulos, H. D., & Faller, Y. N. (2016). Internet-delivered Cognitive Behaviour Therapy for Depressive Symptoms: An Exploratory Examination of Therapist

Behaviours and their Relationship to Outcome and Therapeutic Alliance. Behavioural and Cognitive Psychotherapy, 44(6), 625-639.

Another study that suggested that behaviors such as "alliance bolstering" ("That must have been tough for you" or "Nice to hear that you've had a good week") as well as "task prompting" ("I look forward to hearing how you get on with the next module") and psychoeducation ("Feeling exhausted is common in MS and is also a common symptom of depression") all correlated with more positive outcome in an internet treatment for depression.

General structure of messages: Standardized plus individualized elements

A support web-message generally consists of two elements:

1. A relatively standardized part: e.g., a brief "hello" ("Hello Mr. X") plus brief weekly feedback.

2. An individualized part: those parts of the web messages that cannot be standardized, such as responses to questions that participants ask.

How these elements should be constructed is explained in detail below.

The standardized message elements

- "Hello" and weekly feedback

The therapists are notified within the study platform with regard to which patients are due to be contacted within a given week, and which patients are awaiting a response (because they have sent a message).

Weekly messages - Example „Welcome message"

To standardize the procedure and improve feasibility, all weekly messages should be sent on Mondays, regardless of when participants first entered the trial.

The first message you will send to each participant is the "welcome message". This first message serves multiple purposes:

(1) To introduce yourself
(2) To explain the purpose of the message support ("…to give you feedback and provide support…")
(3) To set realistic expectations
(4) To engage participants, by asking them to describe their personal situation, particularly with regard to MS

English

Dear PARTICIPANT NAME,

Thank you for participating in our study and using the online program. My name is YOUR LAST NAME HERE and I'm a psychologist and part of the study team here at YOUR AFFILIATION HERE.

Please don't hesitate to contact me if you have any questions, comments or concern. I'll always try to respond to you within three days at the most. In addition, I'll be able to track how often you use the program, how you responded to the questionnaires in the program, and which parts of the program you've already seen. I'll send you a brief message every week to give you feedback on your work and support you. I'll generally try to send you my weekly messages on Mondays.

To get started, it would help me to know a few details about your personal situation. Could you briefly describe your personal journey, since you first experienced MS symptoms? It would be helpful for me to know when you first learned about your MS diagnosis, what type or MS you've been diagnosed with, and what you hope to get out of using deprexis? Everything you write to me will be treated confidentially, of course.

I look forward to supporting you over the next weeks and hope you're off to a good start.

Best wishes – YOUR NAME

German:

Liebe/r TEILNEHMER NAME,

Vielen Dank für Ihre Teilnahme bei unserer Studie und Ihrer Bereitschaft, das online Programm zu nutzen. Mein Name ist {HERR/FRAU NACHNAME}, ich bin Psychologe/in und Studienmitarbeiter an der {Charité Universitätsmedizin in Berlin}, von wo aus die Studie geleitet wird.

Sie können mir gerne jederzeit eine Nachricht senden, wenn Sie Fragen, Anmerkungen oder Bedenken bezüglich der Studie oder der Programmnutzung haben. Ich werde versuchen, in spätestens drei Tagen auf Ihre Nachricht zu reagieren. Weiterhin werde ich verfolgen können, wie häufig Sie deprexis nutzen, welche Angaben Sie in den Fragebögen gemacht haben, und welche Programmabschnitte Sie bereits bearbeitet haben. Ich werde Ihnen einmal pro Woche eine kurze Nachricht senden, um Ihnen Feedback zu geben und Sie bei der Beschäftigung mit dem Programm zu unterstützen. In der Regel werde ich versuchen, diese wöchentlichen Rückmeldungen immer am Montag zu senden.

Zum Anfang würde es mir helfen, etwas mehr über Ihre persönliche Situation zu erfahren. Könnten Sie mir deshalb kurz berichten, wie es Ihnen ergangen ist, seit Sie zum ersten Mal MS-Symptome bemerkten? Es wäre auch hilfreich für mich zu wissen, seit wann Sie an MS erkrankt sind, welche Art von MS festgestellt wurde, und welche Hilfe Sie sich von der Beschäftigung mit dem Programm erhoffen. Alles was Sie mir schreiben wird selbstverständlich streng vertraulich behandelt.

Ich freue mich darauf, Sie in den nächsten Wochen zu unterstützen und hoffe, dass sie einen guten Start mit dem Online-Programm haben.

The participant will then be notified via normal personal e-mail that "a message has been received" within the secure online study platform.

If the person decides to respond to your message, you will be notified in the section "patients waiting for a response". You can answer immediately but should definitely answer within three days.

For example, the patient might answer something like the following:

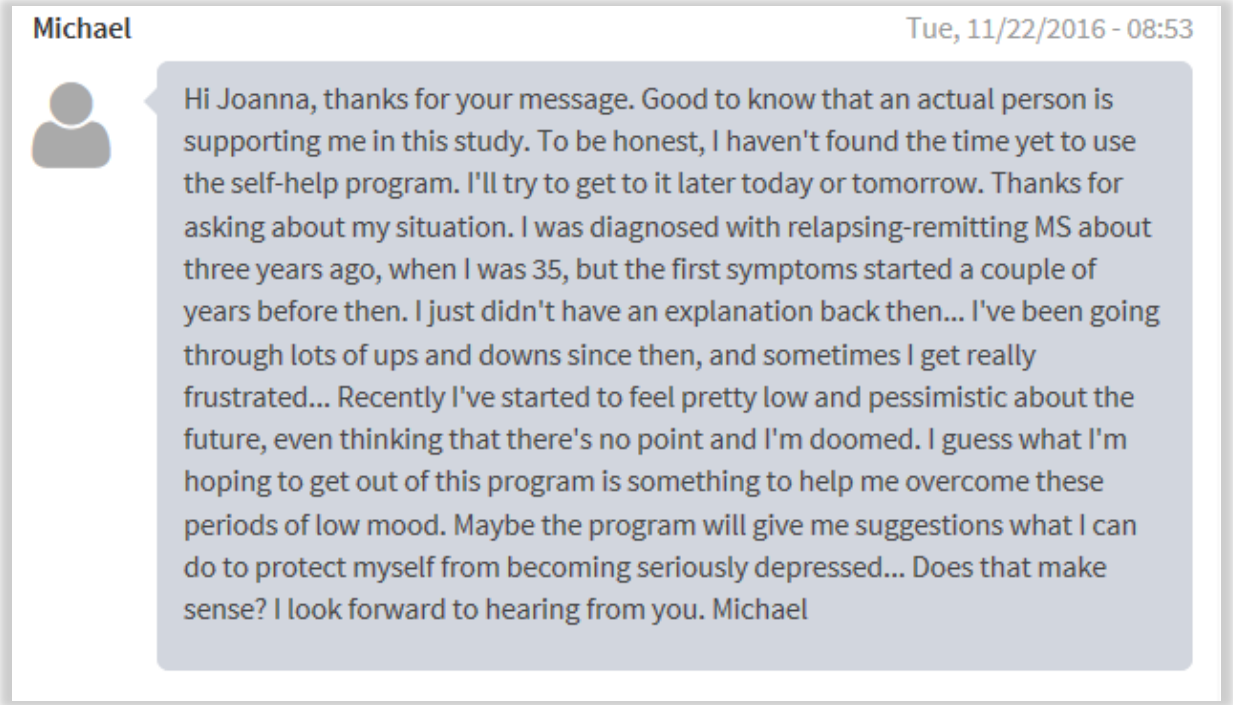
Michael Tue, 11/22/2016 - 08:53

Hi Joanna, thanks for your message. Good to know that an actual person is supporting me in this study. To be honest, I haven't found the time yet to use the self-help program. I'll try to get to it later today or tomorrow. Thanks for asking about my situation. I was diagnosed with relapsing-remitting MS about three years ago, when I was 35, but the first symptoms started a couple of years before then. I just didn't have an explanation back then... I've been going through lots of ups and downs since then, and sometimes I get really frustrated... Recently I've started to feel pretty low and pessimistic about the future, even thinking that there's no point and I'm doomed. I guess what I'm hoping to get out of this program is something to help me overcome these periods of low mood. Maybe the program will give me suggestions what I can do to protect myself from becoming seriously depressed... Does that make sense? I look forward to hearing from you. Michael

You can use your judgment how to respond to this question. Here are two things to keep in mind:

(1) Don’t respond immediately. Wait at least one day (except in emergencies) before you respond. Responding immediately carries that risk that patients come to expect quick responses and will tend to engage in a high-frequency chat-dialogue, which is not the purpose of the web-message support.
(2) When formulating your response, use some of the principles outlined above: Encourage the patient, affirm and validate what they write, prompt program use, and don’t hesitate to explain relevant points briefly (psycho-education).

In this case, this might be an appropriate response (see next page):

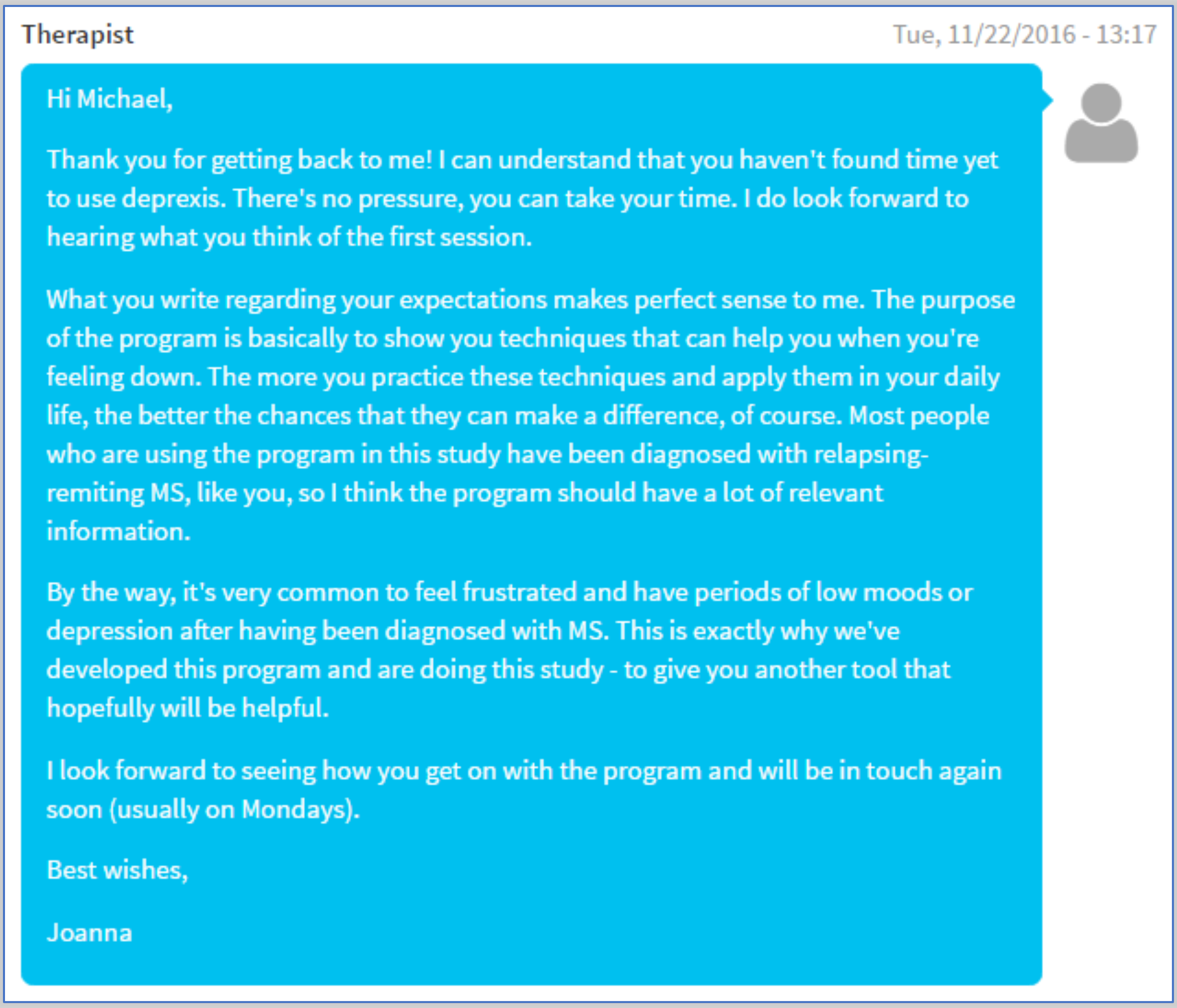

Therapist Tue, 11/22/2016 - 13:17

Hi Michael,

Thank you for getting back to me! I can understand that you haven't found time yet to use deprexis. There's no pressure, you can take your time. I do look forward to hearing what you think of the first session.

What you write regarding your expectations makes perfect sense to me. The purpose of the program is basically to show you techniques that can help you when you're feeling down. The more you practice these techniques and apply them in your daily life, the better the chances that they can make a difference, of course. Most people who are using the program in this study have been diagnosed with relapsing-remiting MS, like you, so I think the program should have a lot of relevant information.

By the way, it's very common to feel frustrated and have periods of low moods or depression after having been diagnosed with MS. This is exactly why we've developed this program and are doing this study - to give you another tool that hopefully will be helpful.

I look forward to seeing how you get on with the program and will be in touch again soon (usually on Mondays).

Best wishes,

Joanna

Writing weekly feedback messages

If possible, the weekly feedback should be sent regularly every Monday. If the participant happens to ask a question around this time, then the response to that question can be combined with the weekly feedback.

The feedback should generally have the following elements (see below). However, individual circumstances might require you to add or omit some parts, as you deem appropriate. You should not always write exactly the same words or sentence. Even the order of these elements does not have to follow a rigid, inflexible pattern; use your own clinical judgment.

1. Brief hello (e.g., “Hello Michael,”)
2. Acknowledge and reinforce effort. Examples:
    - “It was great to see that you worked through the first two chats!”
    - “I saw that you spent a lot of time with the program this week. That’s really good!”
    - “Congratulations! You’ve now completed eight of the chats – that’s quite an accomplishment!”
3. Acknowledge that they may not have used the program and encourage use. Example:

- “I noticed that you did not have a chance to work with the online program this week – that’s perfectly fine! I also hope that you were able to use some of the ideas and techniques from previous chats in your daily life, though!”

4. Provide feedback regarding mood/symptom course (optional, or if you notice something unusual in the mood/symptom trajectory).

    - For example:

        - “I was happy to see that your mood really improved over the last few weeks!”

        - It might be useful to ask questions that encourage reflection: “Why do you think that might be? What was it that made a positive difference?”

        - Another example: I saw that your mood went down quite a bit. What do you think might be the reasons for that? Don’t hesitate to write to me if there’s any way I can support you.

5. Provide feedback regarding the content of the completed chats and emphasis on importance of continuous practice (check which chats have already been completed; if needed, refer to the attached overview of topics).

    - For example: “I hope that the chat about the relationship between negative thoughts and depression was helpful! It’s important to try to notice these thoughts now, and to challenge them. The worksheet on identifying and challenging negative thoughts could help you practice this.”

    - Alternatively, if a patient never writes anything proactively, you could ask a question to engage them: “How did you get on with the relaxation exercises? Were you able to do them and possibly try them out in your daily life?”

6. Remind the patient about the next chat.

    - For example: “You can start with the next chat if you’d like. This one will deal with negative thoughts, and how they can sometimes maintain depressive moods.”

7. The last point to note in your weekly message is that you’re available for questions.

    - For example: “Please don’t hesitate to get in touch with me if you have any questions or comments” or “If you have any questions or concerns, I’ll be happy to help you!”

Example of Complete Message

Combining all the elements described above, a complete weekly message could look like this, for example:

Dear XXX,

It was great to see that you worked through the first two chats, and that your mood seems to have improved a little! I hope you found the relaxation exercises useful got something out of the chat dealing with negative thoughts. It'll be important now to apply the techniques you learned about in your daily life. For example, you could try to identify and challenge negative thoughts you notice. The worksheet might help you with that. You can find this worksheet in your area in the program's menu. Whenever you're ready, feel free to get started with the next chat, which focuses more on acceptance and learning to maintain a relaxed attitude.

Please don't hesitate to contact me if you have any questions or comments about anything. Best wishes, YOUR NAME HERE

MS-specific feedback and responses to queries

- The general suggestion is NOT to give specific advice regarding which medication to take or not to take, which other treatment to pursue, etc.
- Therapists should validate the concern or question and encourage the patient to discuss any specific aspects of disease management with their doctor or treatment team.
- In case of other specific questions, it's recommended to consult with the study team, particularly with Rona Moss Morris.

Statistical Analysis Plan

Version: 1.0 Date: 16.07.2021

International Deprexis Trial in Multiple Sclerosis (IDEMS) – a multicenter randomized controlled trial

IDEMS

**Study Protocol**	Version 1.1 / 19.02.2017
**Clinicaltrials.gov-No.**	NCT02740361
**Internal Protocol ID No.**	Internal protocol number at the CTU-UMG
**NCT-No.**	NCT02740361
**SAP Version**	1.0 / 25.06.2021
**Development Phase**	Phase III
**Coordinating Center**	Charité – Universitätsmedizin Berlin<br>NeuroCure Clinical Research Center (NCRC)<br>Charitéplatz 1, 10117 Berlin
**Coordinating Investigator**	Prof. Dr. Stefan M. Gold<br>Department of Psychiatry and Psychotherapy, Campus Benjamin Franklin<br>Charité – Universitätsmedizin Berlin<br>Charitéplatz 1, 10117 Berlin

Approval of the Statistical Analysis Plan

International Deprexis Trial in Multiple Sclerosis (IDEMS) – a multicenter randomized controlled trial

**NCT No.:**	NCT02740361
**Protocol Version No:**	1.0 / 16.07.2021

Prof. Dr. Stefan Gold	19.7.2021<br>Date	Signature
Biostatistician<br>Prof. Dr. Tim Friede	09 AUG 2021<br>Date	Signature

Table of Contents

Statistical Analysis Plan **1**
Approval of the Statistical Analysis Plan **2**
Table of Contents **3**
List of Figures **4**
List of Tables **4**
List of Abbreviations **5**
1 Introduction **6**
1.1 Background and Rationale 6
1.2 Objective and endpoints 6
1.3 Primary objective and endpoint 7
1.4 Potential moderators 7
2 Study methods **7**
2.1 Trial design 7
2.2 Randomization 8
2.3 Sample Size 8
2.4 Framework 8
2.5 Statistical Interim Analyses and Stopping Guidance 8
2.6 Timing of the Final Analysis 9
2.7 Timing of Outcome Assessments 9
3 Statistical Principles **9**
3.1 Confidence intervals and p-values 9
3.2 Adherence and protocol deviations 9
3.3 Analysis populations 9
4 Trial population **10**
4.1 Screening data 10
4.2 Eligibility 10
4.3 Recruitment 10
4.4 Withdrawal/follow-up 10
4.5 Baseline patient characteristics 10
5 Analysis **11**
5.1 Outcome definitions 11
5.2 Analysis methods 12
5.3 Missing data 13
5.4 Harms 13
5.5 Statistical software 14
5.6 References 14

List of Figures

Figure 1: Trial Design Schematic and Treatment Plan. 8

List of Tables

Table 1: Objectives and related endpoints 6

List of Abbreviations

AE	Adverse Event
ANCOVA	Analysis of Covariance
BDI-II	Beck Depression Inventory – II
BICAMS	Brief Cognitive Assessment in MS
BVMT-r	Brief Visuospatial Memory Test-revised
CFS	Chalder Fatigue Scale
CI	Confidence Interval
CVLT-II T1-5	California Verbal Learning Test-II
EDSS	Expanded Disability Status Scale
FAS	Full Analysis Set
FSMC	Fatigue Scale for Motor and Cognitive
ITT	Intention-To-Treat
LOCF	Last Observation Carried Forward
MADRS	Montgomery-Asberg Depression Rating Scale
MDD	Major Depressive Disorder
MI	Multiple Imputation
M.I.N.I.	Mini-International Neuropsychiatric Interview
mITT	modified Intention-To-Treat
MS	Multiple Sclerosis
MSIS-29	Multiple Sclerosis Impact Scale-29
SAE	Serious Adverse Event
SAP	Statistical Analysis Plan
SBQ-R	Suicide Behaviors Questionnaire – Revised
SDMT	Symbol Digit Modalities Test
SS	Safety Set
SUSAR	Suspected Unexpected Serious Adverse Event
WHO	World Health Organization
WHO-QoL BREF	World Health Organization Quality of Life BREF
WLC	Waitlist Control Group
PDDS	Patient Determined Disease Steps

1 Introduction

This document has been written based on information contained in the trial protocol version 1.1, 02/2017. It describes the analysis after the first 12 weeks. The analysis after 12 months will be listed in a second Statistical Analysis Plan (SAP).

1.1 Background and Rationale

Depression is the most common comorbidity of Multiple Sclerosis (MS). However, depression remains underdiagnosed and there are no treatments with proven effectiveness from large (phase III) trials. In addition, symptoms of MS such as mobility issues, cognitive impairment and fatigue make it difficult for MS patients to travel to and attend regular psychotherapy. Effective treatment options are therefore needed. The internet-based program “Deprexis”, developed by GAIA group in Hamburg/Germany could facilitate access to treatment and has shown promising results in a small monocentre study in Germany for MS patients (1). Deprexis is an online tool and consists of 10 sequential modules plus an introduction and a summary module. Deprexis implements the technique of simulated dialogue by giving the user multiple choice options and tailoring the subsequent content to the patient’s responses. Thereby, the user’s responses determine the course of each module. The Deprexis program has been adapted from the generic version to improve suitability for use by patients with MS. This trial evaluates the effectiveness of MSDeprexis in an international multicentre trial. There are three treatment groups, one group uses only MSDeprexis for 12 weeks, the other group uses MSDeprexis with an added email support for 12 weeks and the third group is a waitlist control group (WLC).

1.2 Objective and endpoints

Table 1 Objectives and related endpoints

	**Objective**	**Endpoint**	**Measurement time points**
**Primary**	Assessment of **depressive symptoms** after intervention	Beck Depression Inventory – II (BDI-II)	V1 (baseline), I1 (after 4 weeks), I2 (after 8 weeks), V2 (after 12 weeks)
**Secondary**	Assessment of **quality of life** after intervention	World Health Organization Quality of Life BREF (WHO-QoL BREF) and the Multiple Sclerosis Impact Scale (MSIS-29) after 12 weeks	V1 (baseline), V2 (after 12 weeks)
	Assessment of **fatigue** after intervention	Fatigue Scale for Motor and Cognitive Functions (FSMC) and the Chalder Fatigue Scale (CFS) after 12 weeks	V1 (baseline), V2 (after 12 weeks)
	Assessment of the **percentage** of patient with a clinical diagnosis	M.I.N.I. structural clinical interview after 12 weeks	V1 (baseline), V2 (after 12 weeks)

	Objective	Endpoint	Measurement time points
	of current **major depressive disorder (MDD)** after intervention		
	Assessment of **severity of depression** after intervention	Clinician-rated Montgomery-Asberg Depression Rating Scale (MADRS) after 12 weeks	V1 (baseline), V2 (after 12 weeks)
**Safety**	Assessment of suicidal ideation	Suicide Behaviors Questionnaire – Revised (SBQ-R), predefined criterion for acute risk of suicide is response 3a or 3b on SBQ-R item 3 plus a score of 5 or 6 on SBQ-R item 4	V1 (baseline), V2 (after 12 weeks)

1.3 Primary objective and endpoint

To determine if MSDeprexis is effective for reducing **depressive symptoms** at the end of treatment (week 12), the Beck Depression Inventory – II (BDI-II)) in patients with MS is used and compared to the control group. Because the literature strongly suggests that therapist-guided internet-based interventions lead to better outcome MSDeprexis with an added email support (MSDeprexisPlus) is also compared to the control group after 12 weeks (MSDeprexis vs. WLC and MSDeprexisPlus vs. WLC).

1.4 Potential moderators

The level of disability measured by the Patient Determined Disease Steps (PDDS), the cognitive impairment measured by Symbol Digit Modalities Test (SDMT) and the level of fatigue measured by the FSMC will be investigated as potential moderators.

2 Study methods

2.1 Trial design

IDEMS is a three arm, randomized, controlled, international multicentre trial with five academic centres. Patients are randomized to one of the three treatment groups after informed consent. One group uses only MSDeprexis for 12 weeks, the other group uses MSDeprexis with an added email support for 12 weeks and the third group is a waitlist control group.

The potential of a booster session to enhance maintenance is analysed in patients who were randomized to either the MSDeprexis or the MSDeprexisPlus group. These patients will be re-randomized to receive additional booster session (see Figure 1). This analysis is not part of this SAP.

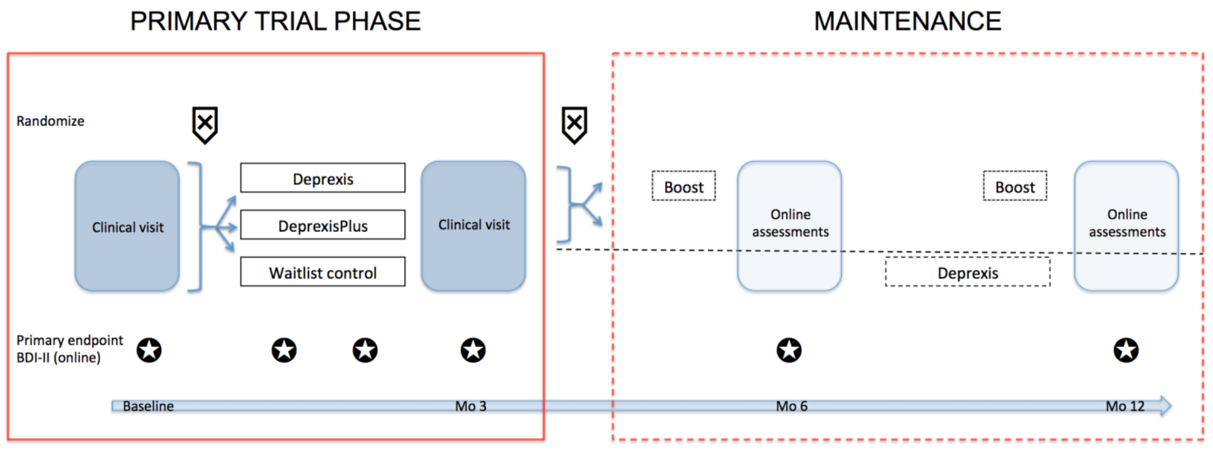

Figure 1: Trial Design Schematic and Treatment Plan.

2.2 Randomization

Patients are randomized 1:1:1 to one of the three trial arms. There is no blocking and no stratification. To ensure concealed allocation, eligibility is determined and all baseline assessments completed before randomization in compliance with CONSORT guidelines. The clinicians and raters who will be conducting clinical assessments (structured interviews) will be blind to treatment assignment (single blind RCT).

2.3 Sample Size

A sample size of 100 patients per intervention group gives a conjunctive power (probability of rejecting both null hypotheses comparing MSDeprexis and MSDeprexisPlus to waitlist control) of 90% for a Dunnett test at the usual one-sided significance level of 2.5% assuming standardized mean differences of 0.5 for MSDeprexis vs. WLC and 0.8 for MSDeprexisPlus vs. WLC in the primary outcome change in BDI from baseline to week 12. Adjusting for 20% dropout we aim to recruit 125 patients per group resulting in a total sample size of 375 patients. The power was simulated with 10,000 replications using EAST 6.3.

2.4 Framework

The superiority of MSDeprexis compared to a waitlist control group is analysed. Specifically, the use of MSDeprexis only as well as the use of MSDeprexis with an added email support are compared to the control group.

2.5 Statistical Interim Analyses and Stopping Guidance

No interim analyses or guidelines for stopping the trial early are planned. There are no predefined stopping rules.

2.6 Timing of the Final Analysis

All outcomes are analysed collectively after the last patient has completed the visit after 12 weeks. In addition, to explore the long-term stability of therapeutic effects and the potential of a booster session to enhance maintenance another evaluation takes place after 12 months (not part of this SAP).

2.7 Timing of Outcome Assessments

Outcomes are measured at week 0 (baseline) and week 12 when visiting the clinic. In addition, BDI-II outcomes are measured at week 4 and 8 in online assessments. Follow-up visits for all patients who want to use MSDeprexis after 6 and 12 month are conducted online.

3 Statistical Principles

3.1 Confidence intervals and p-values

If not specified otherwise, tests will be performed two-sided with a significance level of 5% and 95% confidence intervals (CI) will be provided for parameter estimates. For the primary analysis, MSDeprexis vs. WLC and MSDeprexisPlus vs. WLC will be tested by a Dunnett test controlling the familywise type I error rate at the level of 2.5% (one-sided).

3.2 Adherence and protocol deviations

The number of days with activity in the MSDeprexis programm by each patient is assessed as a measure of treatment adherence.

3.3 Analysis populations

Full analysis set (FAS)

The FAS is based on the intention-to-treat (ITT) principles. This means that all randomized patients with at least one post-baseline assessment will be included in the analysis. A modified ITT (mITT) population will be analysed in a sensitivity analysis. It includes those patients who have registered in the program MSDeprexis.

Safety Set (SS)

All subjects who have registered in the program MSDeprexis will be included. Subjects will be analysed according to the treatment they received.

4 Trial population

4.1 Screening data

Screening takes place within the participating centres. Available data on patients assessed for eligibility will be included within a CONSORT flow diagram.

4.2 Eligibility

Patients need to be at least 18 years old with a neurologist-confirmed diagnosis of MS. They have self-reported depressive symptoms (BDI-Fastscreen ≥ 4) and are able to travel to the outpatient centre for two clinical assessments. Acute risk for suicide at baseline will lead to exclusion from the trial. For complete inclusion/exclusion criteria, see section 4 of the latest version of the study protocol.

4.3 Recruitment

A CONSORT flow diagram will be calculated to show patient disposition including sample sizes for recruitment, randomization and analyses.

4.4 Withdrawal/follow-up

Withdrawal rates will be calculated and reasons given within a CONSORT flow diagram.

4.5 Baseline patient characteristics

Baseline characteristics and demographic data will be summarized descriptively stratified by treatment group:

- Age [years]
- Sex [female; male]
- Marital status [married of domestic partnership; separated or divorced; single, never married; widowed]
- Education [high school graduate, diploma or equivalent; associate degree, bachelor's degree; master's degree; doctoral degree]
- Employment status [unemployed or retired; homemaker; student, parttime; full time; other]
- Time since diagnosis
- Diagnosis subtype [primary progressive MS; secondary progressive MS; relapsing-remitting MS; unclear]
- Neurological status [Expanded Disability Status Scale (EDSS)]
- Level of disability [PDDS]
- Neurological function [BICAMS]

- Disease-modifying therapy.

5 Analysis

5.1 Outcome definitions

Depressive symptoms at baseline and at week 4, 8 and 12 (end of treatment) are measured with the BDI-II. BDI-II is a 21-question self-reported inventory for measuring the severity of depression. Each question has a set of at least four possible responses, ranging in intensity. To calculate the score a value of 0 to 3 is assigned for each answer. A higher total score indicates more severe depressive symptoms. The interpretation of these scores is:

- 0-13: minimal depression
- 14-19: mild depression
- 20-28: moderate depression
- 29-63: severe depression (2)

Quality of life is measured at baseline and at week 12 with the WHO-QoL BREF and with the MSIS-29. WHO-QoL BREF consists of 26 items, which are assigned to the four domains: physical health, psychological health, social relationships, and environment. Answers are on a 5-points Likert scale ranging from 1 (not at all) to 5 (completely). Items are scored from 1 to 5 and a score (0-100) is calculated using an algorithm (see Appendix 10 of (3)).
The Multiple Sclerosis Scale-29 includes two scales: physical impact (20 items) and psychological impact (nine items). All items have five response options from 1 (not at all) to 5 (extremely). A score (0-100) is calculated using the following algorithm: Physical scale items (1-20): sum, subtract 20, divide by 80, and multiply by 100, psychological scale items (21-29): sum, subtract nine, divide by 36, and multiply by 100 (4).

Fatigue is measured at baseline and at week 12 with the FSMC and the Chalder Fatigue Scale. FSMC is a 20-item scale with response option from 1 (Does not apply at all) to 5 (Applies completely). There are ten questions about the cognitive and ten questions about the motor fatigue, the total score can reach 100 points (extreme fatigue) (5).
The Chalder Fatigue Scale is an 11-item questionnaire with a 4-points Likert scale ranging from 0 (Less than usual) to 3 (Much more than usual). The sum of these items is the score, so it can range from 0 to 33 (6).

In addition, depressive symptoms are measured with the Mini-International Neuropsychiatric Interview (M.I.N.I.) and the MADRS. M.I.N.I. is a short structured diagnostic interview for the major Axis I psychiatric disorders in DSM-IV and ICD-10 (7).
MADRS is a ten-item questionnaire with a 7-points Likert scale from 0 to 6. The score is the sum of all items and ranges from 0 to 60 (8).

For safety analysis the SBQ-R is used to control suicidal ideation. The SBQ-R has 4 questions, where each question has an individual scale, and each response corresponds to a certain point value. The total score ranges from 3 to 18. The higher the score, the higher the likelihood of suicidal behavior (9).

To determine the neurological function the Symbol Digit Modalities Test (SDMT) is used. This test is part of the Brief Cognitive Assessment in MS (BICAMS). The SDMT consists of a row with nine symbols, where each symbol corresponds to a certain value. Below there are seven rows with 15 symbols each and the patient should assign the correct numerical value to as many symbols as possible within 90 seconds. The score is the number of correct assignments. There are two additional tests: The California Verbal Learning Test-II (CVLT-II T1-5). There is a 16-item list with words from four different topics, arranged randomly. The list is read aloud five times in the same order to the patient. Patients are required to recall as many items as possible, in any

order, after each reading of the list. The other one is the Brief Visuospatial Memory Test-revised (BVMT-r). In this test 6 abstract figures are presented three times. In each of the three learning trials, the patient views the same array for 10 seconds. Then the array is removed and the patient is required to draw the stimulus array from memory, with the correct shapes in the correct position. Each design receives from 0 to 2 points representing accuracy and location, so the score ranges from 0 to 12 for each trial (10, 11).

To determine the neurological impairment the Patient Determined Disease Steps (PDDS) is used. There are 9 categories from 0 (no limitation of activities) to 8 (bedridden) describing the patients situation with a main focus on the ability to walk (12).

5.2 Analysis methods

Primary endpoint

The primary outcome change in BDI from baseline to week 12 will be analysed by means of linear mixed effects models for repeated measures adjusted for baseline measurements with fixed effects for intervention, region (US, GER), time and baseline BDI score, and random subject effects for individual patients including all patients with at least one post-baseline measurement (13). Least squares means will be reported for the intervention groups with 95% CI as well as the difference between the least squares group means with 95% CI. MSDeprexis vs. WLC and MSDeprexisPlus vs. WLC will be tested by a Dunnett test controlling the familywise type I error rate at the level of 2.5% (one-sided). The primary hypothesis will be tested in a confirmatory manner:

$H_0^{(1)}: \mu_{WLC} \leq \mu_{Dep}$ vs. $H_1^{(1)}: \mu_{WLC} > \mu_{Dep}$ and $H_0^{(2)}: \mu_{WLC} \leq \mu_{DepPlus}$ vs. $H_1^{(2)}: \mu_{WLC} > \mu_{DepPlus}$,
with μ_{WLC}, μ_{Dep} and $\mu_{DepPlus}$ being the means of the change in BDI from baseline to week 12.

In a secondary step, the added value of therapist email support MSDeprexis vs. MSDeprexisPlus at a two-sided level of 5%, if efficacy of MSDeprexis and MSDeprexisPlus for reducing depressive symptoms in MS could be demonstrated, will be determined.

SAS analysis:

```
proc mixed data =_data_t;
class group time region;
model score = group time group*time baseline baseline*time region / s ddfm=kr;
repeated time / subject=id type=un;
lsmeans group / adjust = dunnett;
```

In a sensitivity analyses last observation carried forward (LOCF) and multiple imputations (MI) will be used to deal with missing values for the BDI-II score (missing visits) and for each method an analysis of covariance (ANCOVA) for the BDI-II score after 12 weeks is carried out with BDI-II score at baseline as covariate.

In an exploratory analysis trends in the utilization time of MSDeprexis [number of days with activity within the program] in both intervention groups will be investigated.

Secondary endpoints

The analyses of secondary endpoints QoL (WHO-QoL and MSIS-29), fatigue (FSMC and CFS) and the severity of depression (MADRS) will follow the similar approach as the analyses described for the primary endpoint. The psychological score of the WHO-QoL and the FSMC are defined as the key secondary endpoints.

The number of patient with a clinical diagnosis of current major depressive disorder (M.I.N.I.) will be analysed using a logistic regression model with the variables treatment group and baseline score of BDI-II.

Potential moderators

The potential moderators (SDMT, PDDS and FSMC) will be investigated in a supporting exploratory analyses by including these and their interaction with treatment in the linear mixed effects models described above.

Safety analyses

The analysis of suicidal ideation (measured by SBQ-R) is done analogously to the primary endpoint.

5.3 Missing data

Five patients have no online baseline assessment (technical problems). For the primary analysis these missing baseline BDI-II scores will be replaced by a regression imputation with the MADRS scores which were collected paper based. Although the mixed model described above is robust to a certain extent to missing data, sensitivity analyses will be performed as supporting analyses (see section 5.2).

5.4 Harms

Adverse events will be summarized as frequencies and percentages by intervention group. New occurrence of suicidal ideation or intent as well as worsening of depressive symptoms above the clinical threshold are considered as potential adverse events. Every AE fulfilling one of the following criteria is a SAE:

- Suicidal ideation or intent (as measured by a score of 3 on BDI-II item 9 at any assessment, during the clinical interview at V2 or spontaneous report in a web message to the therapist or by contacting the study site)
- Hospitalization due to psychiatric disorder classified according to ICD10 or DSM5
- Lethal or life-threatening (incl. suicide or suicide attempt).

However, the intervention is generally considered low risk as Deprexis has been used in numerous clinical trials without evidence for adverse events and is categorized as a “low risk” medical device according to its German CE®-certification.

5.5 Statistical software

Statistical programming will be done using R version 4.0.0 or higher and SAS version 9.4 or higher.

5.6 References

1. Fischer, A., Schröder, J., Vettorazzi, E., Wolf, O. T., Pöttgen, J., Lau, S., ... & Gold, S. M. (2015). An online programme to reduce depression in patients with multiple sclerosis: a randomised controlled trial. *The Lancet Psychiatry*, *2*(3), 217-223.
2. Beck, A. T., Steer, R. A., & Brown, G. K. (1996). *Manual for the Beck depression inventory-II*. San Antonio, TX: Psychological Corporation.
3. World Health Organization (1998). Programme on metal health – WHOQOL User Manual.
4. Widener, G. L., & Allen, D. D. (2014). Measurement characteristics and clinical utility of the 29-item Multiple Sclerosis Impact Scale. *Archives of physical medicine and rehabilitation*, *95*(3), 593-594.
5. Penner, I. K., Raselli, C., Stöcklin, M., Opwis, K., Kappos, L., & Calabrese, P. (2009). The Fatigue Scale for Motor and Cognitive Functions (FSMC): validation of a new instrument to assess multiple sclerosis-related fatigue. *Multiple Sclerosis Journal*, *15*(12), 1509-1517.
6. Jackson, C. (2015). The Chalder fatigue scale (CFQ 11). *Occupational Medicine*, *65*(1), 86-86.
7. Sheehan, D. V., Lecrubier, Y., Sheehan, K. H., Amorim, P., Janavs, J., Weiller, E., Hergueta, T., Baker, R., & Dunbar, G. C. (1998). The Mini-International Neuropsychiatric Interview (M.I.N.I): The development and validation of a structured diagnostic psychiatric interview for DSM-IV and ICD-10. *The Journal of Clinical Psychiatry, 59*(Suppl 20), 22–33.
8. Montgomery, SA (1979). Åsberg M. *A new depression scale designed to be sensitive to change. Br J Psychiatry*, *134*, 382-389.
9. Osman, A., Bagge, C. L., Gutierrez, P. M., Konick, L. C., Kopper, B. A., & Barrios, F. X. (2001). The Suicidal Behaviors Questionnaire-Revised (SBQ-R): validation with clinical and nonclinical samples. *Assessment*, *8*(4), 443-454.
10. Langdon, D. W., Amato, M. P., Boringa, J., Brochet, B., Foley, F., Fredrikson, S. & Reder, A. T. (2012). Recommendations for a brief international cognitive assessment for multiple sclerosis (BICAMS). *Multiple Sclerosis Journal*, *18*(6), 891-898.
11. Langdon, D., Amato, M., Boringa, J., Brochet, B., Foley, F., Fredrikson, S. & Reder, A. (2011). The Brief International Cognitive Assessment for Multiple Sclerosis (BICAMS): first consensus steps towards a brief universal cognitive assessment for MS. In *Neurology* (Vol. 76, No. 9, pp. A479-A479). 530 WALNUT ST, PHILADELPHIA, PA 19106-3621 USA: LIPPINCOTT WILLIAMS & WILKINS.
12. Hohol, M. J., Orav, E. J., & Weiner, H. L. (1995). Disease steps in multiple sclerosis: a simple approach to evaluate disease progression. *Neurology*, *45*(2), 251-255.
13. Mallinckrodt CH, Clark SW, Carroll RJ, Molenbergh G (2003). Assessing response profiles from incomplete longitudinal clinical trial data under regulatory considerations. Journal of biopharmaceutical statistics. 13:179-190.
14. Henderson R, Diggle P, Dobson A (2000). Joint modelling of longitudinal measurements and event time data. Biostatistics. 1:465-480.

International Deprexis Trial in Multiple Sclerosis (IDEMS) – a multicenter randomized controlled trial

Acronym: IDEMS
Protocol Code:
Clinicaltrial.gov identifier: NCT02740361

Protocol

Version 1.1 / Date: 19.02.2017

Coordinating Center: Charité – Universitätsmedizin Berlin
NeuroCure Clinical Research Center (NCRC)
Charitéplatz 1, 10117 Berlin

Principal Investigator: Prof. Dr. Stefan M. Gold
Department of Psychiatry and Psychotherapy, Campus Benjamin Franklin
Charité – Universitätsmedizin Berlin

Biostatistician: Prof. Dr. Tim Friede

We hereby agree on the following protocol. We are aware of the ICH-GCP guidelines and certify the trial will be conducted according to those regulations.

**Principal Investigator**<br>Stefan M. Gold (P.I.)	Signature	3/17/2017 Berlin<br>Date, Place

- Confidential -

The content of the protocol is confidential and serves to inform the following peers about content and course of the trial: investigators, their employees, the ethics committee and competent authority as well as a CRO, monitor and to inform possible participants.

Abbreviations

AE	Adverse Event
CBT	cognitive behavioral therapy
ICBT	internet-based CBT
SAE	Serious Adverse Event
SUSAR	Suspected Unexpected Serious Adverse Event
ITT	Intent to treat Population
PPP	Per protocol population
RCT	Randomized controlled trial
WLC	waitlist controls

1 Synopsis

Study Title	**International Deprexis Trial in Multiple Sclerosis (IDEMS) – a multi-center randomized controlled trial**
**Type of Study**	Randomized controlled trial
**Coordinating Center**	Charité – Universitätsmedizin Berlin<br>NeuroCure Clinical Research Center (NCRC)<br>Charitéplatz 1, 10117 Berlin<br>FAX: +49 30 450 539 921
**Principal Investigator**	Prof. Dr. Stefan M. Gold<br>Responsible medical investigator at coordinating center:<br>Prof. Dr. Friedemann Paul
**Hypotheses**	The online program “Deprexis” is effective in reducing depressive symptoms in patients with multiple sclerosis
**Rationale**	Depression is the most common comorbidity of MS. However, depression remains underdiagnosed and there are no treatments with proven effectiveness from large (phase III) trials. In addition, symptoms of MS such as mobility issues, cognitive impairment and fatigue make it difficult for MS patients to travel to and attend regular psychotherapy. Effective treatment options are therefore needed. The internet-based program “Deprexis” could facilitate access to treatment and has shown promise in a small monocenter study in Germany. The current trial will evaluate the effectiveness of Deprexis in an international multicenter trial.
**IMP & Treatment**	a) Deprexis as stand-alone for 12 weeks<br>b) Deprexis with added email support for 12 weeks
**Control**	Waitlist control
**Study design**	Three arm, randomized, controlled, multicenter trial
**Anticipated schedule**	Initiation of recruitment – February 2017<br>Closure of recruitment – February 2019<br>Closure of trial – September 2019
**Number of participants**	375
**Target Population**	Multiple sclerosis
**Inclusion Criteria**	- age ≥ 18<br>- neurologist-confirmed diagnosis of MS<br>- self-reported depressive symptoms (BDI-Fastscreen ≥ 4)<br>- fluent in German or English (depending on study site),<br>- willingness to engage in self-administration of an iCBT intervention for 12 weeks and complete follow-up<br>- ability to travel to the outpatient center for two clinical assessments (baseline and week 12)<br>- internet access at home<br>- informed consent by patient
**Exclusion Criteria**	- unwilling or unable to consent,<br>- diagnosis of bipolar or psychosis (as determined by M.I.N.I structured interview),<br>- substantial neurocognitive impairments such as dementia or autism<br>- moderate or high risk of suicide (according to MINI module C) or by clinical impression

	- very severe depression that would interfere with the ability to participate in the study (based on clinical judgment by the physician at the recruitment site). Patients with very severe depression will be referred to psychiatric services for immediate treatment.<br>- current psychotherapy/behavioral treatments for depression<br>- started pharmacotherapy for depression within the last 2 months<br>- MS relapse or steroid treatment in the last 4 weeks<br>- concurrent participation in another interventional clinical trial<br>- Refusal to saving, processing and forwarding of pseudonymized data
**Visits**	Clinical visits:<br>Baseline and Week 12<br>Online questionnaires:<br>Month 6 and Month 12
**Endpoints**	• Primary endpoint: Beck Depression Inventory - II<br>• Secondary endpoints:<br>o WHO-QoL BREF<br>o Multiple Sclerosis Impact Scale, MSIS-29<br>o Fatigue Scale for Motor and Cognitive Functions (FSMC)<br>o Chalder Fatigue Scale<br>o Current MDD diagnosis (M.I.N.I. structured clinical interview, clinician-rating, version 5.0.0)<br>o Montgomery Asberg Depression Rating Scale (MADRS)<br>• Safety and moderators<br>o Suicidal Behaviors Questionnaire-Revised<br>o Brief Cognitive Assessment in MS (BICAMS)
**Safety**	Documentation of adverse events with every visit.<br>SOP suicidality
**Criteria for Discontinuation**	Of Participation for an Individual:<br>Obligatory: Personal decision of the individual, potential harm (occurrence of clinically relevant suicidal ideation or at the discretion of the investigator)<br>Possible: Retrospectively assessed exclusion criterion<br>Severe Adverse Event (SAE) or Suspected Unexpected Serious Adverse Reaction (SUSAR)<br>Of the trial itself:<br>Change of the risk-benefit-analysis
**Statistical Evaluation/Sample Size Calculation**	The primary outcome change in BDI from baseline to week 12 will be will be analyzed as intention-to-treat (ITT) by means of linear mixed effect models for repeated measures adjusted for baseline measurements including all patients with at least one post-baseline measurement. A sample size of 100 patients per intervention group gives a conjunctive power (probability of rejecting both null hypotheses comparing Deprexis and DeprexisPlus to waitlist control) of 90% for a Dunnett test at the usual one-sided significance level of 2.5%. Adjusting for 20% dropout we aim to recruit 125 patients per group resulting in a total sample size of 375 patients.
**Pharmacological-toxicological Evaluation**	N/A
**Possible Risks, Adverse Reactions, Contraindications, Procedures in case of incidents**	The intervention is generally considered low risk as Deprexis has been used in numerous clinical trials without evidence for adverse events and is categorized as a "low risk" medical device according to

	its German CE®-certification. We will exclude patients with more than a low risk of suicidality. Standard operating procedures are in place to respond to occurrence of suicidal thoughts and intent in any participant.
**Risk-Benefit-Analysis**	Risk-benefit ratio is considered acceptable as risk is low but patients may benefit from a new online intervention tool to reduce depressive symptoms.

Course of trial (flow chart)

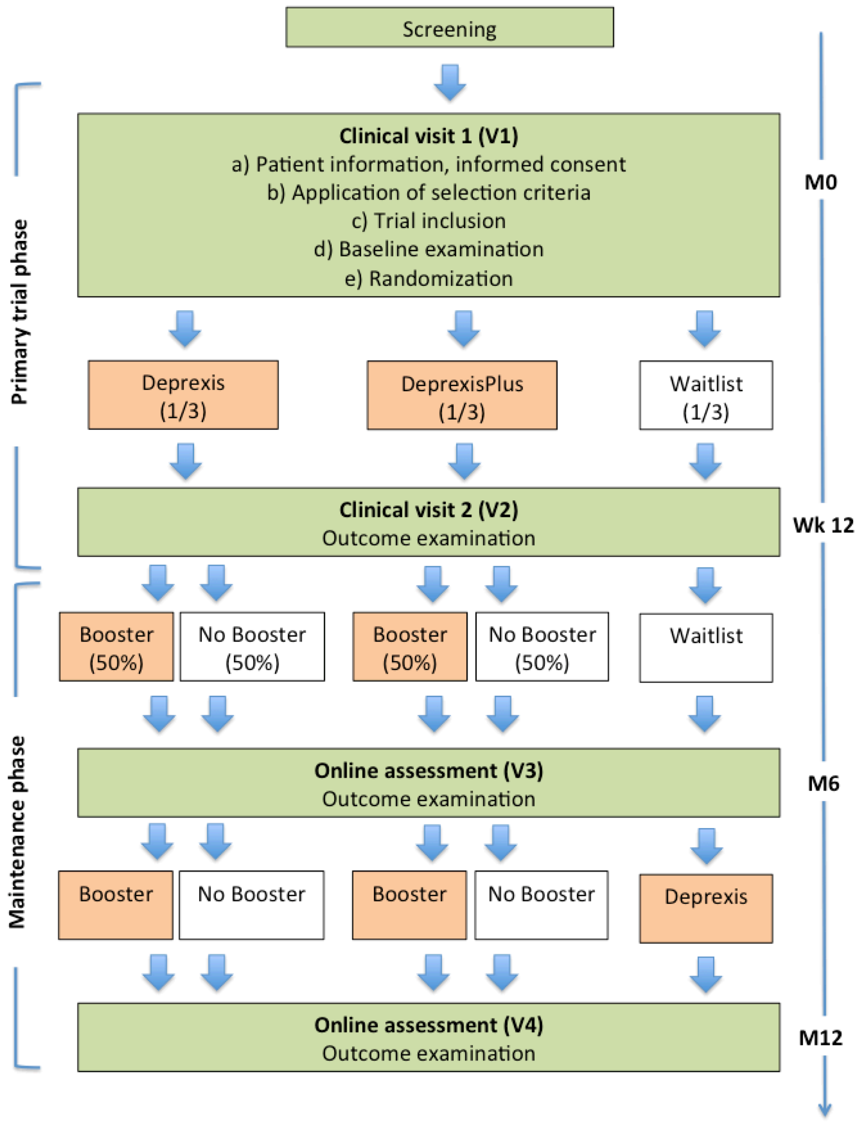

2 Introduction

2.1 Introduction and Background

Prevalence and impact of depression in MS

MS patients frequently experience neuropsychiatric symptoms such as depressive mood, fatigue, and cognitive impairment. Depression is common with a lifetime risk for major depressive disorder (MDD) as high as 25-50% (1) and a point prevalence of up to 25% (2). Depression is particularly frequent in younger patients (3). Depression in MS has been linked to biological as well as psychological factors and substantially impacts psychosocial function (4). Importantly, depressive symptoms correlate with decreased quality of life, absence from work, and lower social support in MS patients (5, 6). Depression is also associated with lower immunotherapy adherence rates and may thus have direct consequences for overall health outcome (7). Moreover, depression is one of the main predictors for suicidal ideation and suicide risk in patients with MS (8). If left untreated, depressive symptoms in MS rarely remit spontaneously, often become chronic (9), and may worsen over time, particularly in patients with baseline scores indicative of clinical depression (10). Despite its immediate clinical relevance, depression remains widely undiagnosed and untreated in MS patients (11).

The need to develop novel therapeutic options for depression in MS

Unfortunately, evidence for the efficacy of pharmacological or non-pharmacological interventions for MS-associated depression is scarce. Only two small placebo-controlled randomized controlled trials (RCTs) to date have evaluated the effects of pharmacotherapy with desipramine or paroxetine in MS-associated depression (12, 13). A Cochrane review (14) concluded that there was some benefit (albeit not statistically significant for most endpoints) but also a risk for adverse side effects such as nausea and headaches. One recent meta-analysis supports the efficacy of cognitive behavioral therapy (CBT) in individual or group settings (SMD -0.46, 95% CI -0.75 to -0.17, p=0.002) to reduce depressive symptoms in MS patients (15). This analysis however also found high levels of statistical heterogeneity. This means that there was substantial variance in the size of the treatment effect between the different trials that was larger than expected by chance alone, reducing the reliability of the aggregate effect size and thereby weakening the conclusions that can be drawn.

Since MS frequently causes motor impairment and decreased mobility as well as increased fatigability and cognitive problems, self-paced, remote access options for psychotherapy may be particularly useful to enhance availability of effective depression interventions such as CBT for MS patients. For example, psychotherapy delivered by phone has been shown to decrease depressive symptoms in MS patients (16, 17). Such approaches, however, still require availability of a trained psychotherapist. Guidelines for psychiatric disorders in MS published by the American Academy of Neurology in 2014 recommended the use of telephone-administered CBT with weak level of evidence (level C) and concluded that evidence for pharmacotherapy and individual and group therapies was insufficient (level U) (18). Thus, there is an urgent, unmet need to develop and rigorously test the efficacy of treatment strategies for MS-associated depression and to facilitate access to these treatments. Importantly, all RCTs for depression treatments in MS – be it pharmacological or behavioral – have been conducted in relatively small samples (range n=19 to n=127). To date, large, definitive trials (phase III) of the most promising therapeutic approaches that could inform clinical practice are completely lacking.

Preliminary Studies

Given the mobility issues and fatigability typically associated with MS as well as the limited availability of psychotherapists, self-guided, automated, internet-based interventions might be particularly useful for MS patients with a need for depression treatment, at least as an interim solution until psychotherapy becomes available. Another obvious advantage of such interventions would be that they can be broken down into smaller modules and completed at any time, therefore allowing patients with increased fatigability or deficits in cognitive domains such as processing speed or attention to proceed at their own pace. Among all available fully-automated, internet based CBT interventions, a program called “Depexis” developed by GAIA group in Hamburg/Germany is one of the most researched options (with 6 published phase II RCTs to date). In addition, a recent meta-analysis suggested that among the fully-automated programs, it is the most effective (19).

Evidence for efficacy of Deprexis

Evidence from several RCTs to date supports the efficacy of Deprexis among adults with elevated depressive symptoms when compared to waitlist control. In a first trial (20), n=396 participants were recruited from Internet forums in Germany and randomized on a 4:1 schedule to Deprexis or a waitlist control condition. In this trial, a between-group posttreatment effect size of d=0.65 (linear mixed-model analyses) was observed. However, attrition was somewhat problematic in this study (only 55% of participants completed posttreatment assessments). Another RCT with n=210 participants (21) confirmed a significant treatment effect of Deprexis, albeit with a smaller effect size (between-group difference d=0.36). Here, attrition was acceptable, with 82% completing the post-assessment. Another trial explored the program's efficacy among patients with epilepsy and elevated levels of depressive symptoms (22). In a sample of n=78 patients, the self-guided Deprexis version yielded small effects (between-group difference d=0.46). Attrition rate was in an acceptable range, with 72% completing posttreatment assessments. Finally, one recent RCT (n=163) supports the program's efficacy among adults with initially severe depression symptoms (23). Here, a posttreatment between-group effect of d=0.57 was observed. Attrition rates were acceptable, with 81% of participants completing post-assessments.

Study name	Outcome / Population	Statistics for each study		
		Hedges's g	Lower limit	Upper limit
Meyer, 2009	BDI / general population / moderate symptoms	0,64	0,33	0,94
Berger, 2011 (guided)	BDI-II / general population / moderate symptoms	1,14	0,55	1,72
Berger, 2011 (unguided)	BDI-II / general population / moderate symptoms	0,72	0,16	1,27
Moritz, 2012	BDI / general population / moderate symptoms	0,43	0,13	0,73
Schröder, 2014	BDI / epilepsy / mild to moderate symptoms	0,22	-0,30	0,73
Fischer, 2015	BDI / multiple sclerosis / mild to moderate symptoms	0,32	-0,09	0,74
Meyer, 2015	PHQ-9 / general population / severe symptoms	0,57	0,22	0,91
		0,54	0,39	0,69

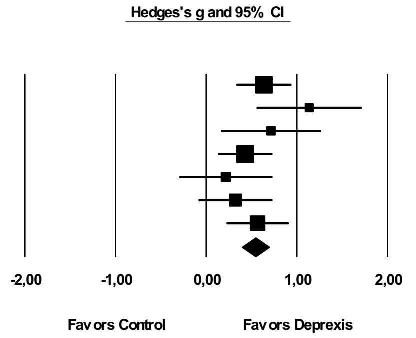

Figure 1: Meta-analytic summary of all published RCTs comparing Deprexis to a waitlist control group (forest plot). Data support efficacy of Deprexis with a medium effect size (p<.001).

The added value of therapist support: Deprexis vs DeprexisPlus

The literature on internet-based interventions strongly suggests that guided internet-based cognitive behavioral therapy (ICBT) is more effective than self-guided treatments and that various types of contact with a clinician or trained technician (like an interview or coming for assessment) tends to lead to better outcomes (24). Although Deprexis has been found to show the largest effect size of any of the fully automated programs evaluated to date, its efficacy might still be enhanced further by adding therapist contact. One small study compared the benefits of Deprexis with the same intervention complemented with weekly therapist support via e-mail (25). A waiting-list control group was also included. Seventy-six individuals meeting the diagnostic criteria of major depression or dysthymia were randomly assigned to one of the three conditions. The Beck Depression Inventory (BDI-II) was used as the primary outcome measure. Secondary outcomes included general psychopathology, interpersonal problems, and quality of life. Sixty-nine participants (91%) completed the assessment at posttreatment and 59 (78%) at 6-month follow-up. Results showed significant symptom reductions in both treatment groups compared to the waiting-list control group. At posttreatment, between-group effect sizes on the BDI-II were d=0.66 for unguided Deprexis versus waiting-list and d=1.14 for guided Deprexis versus waiting-list controls. In the comparison of the two active treatments, small-to-moderate, but not statistically significant, effects in favor of the guided condition were found on all measured dimensions. In both groups, treatment gains were maintained at 6-month follow-up. The findings provide evidence that internet-delivered treatments for depression can be effective whether support is added or not but that the efficacy of interventions such as Deprexis might be further enhanced by email therapist support.

The outcome measures, study populations, and results of all published Deprexis RCTs are summarized in Figure 1, which shows the weighted average effect size derived from reported post-treatment means and standard deviations (two effect estimates from Berger et al. (25), given that a guided and unguided program version were compared). Across these studies, Deprexis was

associated with a medium effect size, Hedges g=0.54, 95% CI: 0.39 to 0.69, p<0.001, with low and non-significant heterogeneity, I^2=14.71%.

Pilot data on US version of Deprexis

An English language version of Deprexis is currently being evaluated in a RCT at the University of Texas Austin (clinicaltrials.gov identifier NCT01818453, PI: Prof. Christopher Beevers, Dept Psychology, Director of the Institute for Mental Health Research). To date, 206 participants with an elevated level of depression are enrolled in the study. Interview based ratings of depression severity were obtained using the Hamilton Depression Rating Scale. There was a significant difference between the Hamilton score at baseline and follow-up (p<0.001). Although these data are based on an interim analysis and the control group has not been analyzed so far, these data indicate that the translated version of Deprexis is similarly effective in a US population and achieves comparable effects to the German version.

Phase II trial of Deprexis in MS

We recently conducted a phase II RCT to test the feasibility and efficacy of the fully automated, internet-based cognitive behavioral therapy (iCBT) program Deprexis to reduce depressive symptoms in MS patients (26). A total of 241 patients were screened and 96 were eligible for the trial. We randomized n=90 of the eligible patients (n=45 Deprexis, n=45 waitlist controls (WLC)) and n=71 completed the study resulting in a dropout rate of 21%. Drop-out was similar in the groups (Deprexis n=10, 22·2%; WLC n= 9, 20%). In the Deprexis group, BDI scores decreased over time, with scores slightly increasing in the WLC group (Figure 2, left panel). ANCOVA intention-to-treat analysis revealed significant treatment effects (mean difference 4·02 (CI 0.79; 7.26); p=0.015) with moderate effect size (d=0.53). Group differences were slightly larger in the mixed models, multiple imputations, and the per protocol sensitivity analyses. The fraction of patients above the cut-off for clinical depression decreased from 68% (n=24) to 54% (n=19) in the Deprexis group. In contrast, the percentage of patients above the clinical cutoff slightly increased from 67% (n=24) to 69% (n=25) in the WLC (see Figure 2, right panel). When analyzed by Cochran Chi-sq, this difference was significant (p=0.01). Based on the BDI categorical analysis, number needed to treat (NNT) was eight.

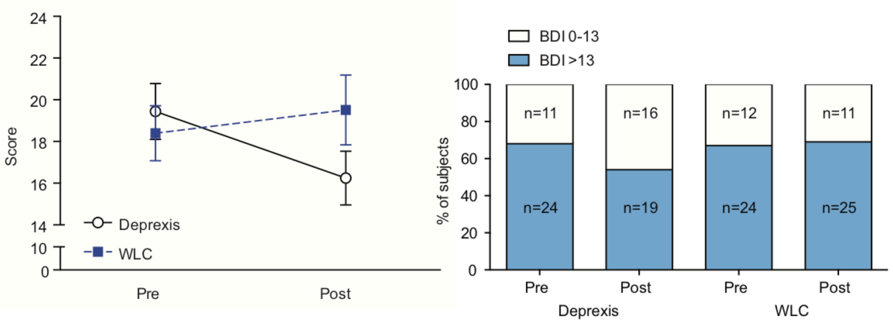

Figure 2: Phase II RCT of Deprexis to reduce depressive symptoms in MS. After the 9-week program, depression scores decrease significantly compared to the waitlist controls (WLC) with moderate effect size (p=0.015; d=0.53, left panel). The proportion of patients with clinically relevant depression (BDI>13) was also significantly reduced by the intervention compared to WLC (p=0.01). Taken from Fischer et al., *Lancet Psychiatry* (2015).

A subgroup of participants (n=34: Deprexis n=17, WLC n=17) from the online cohort volunteered to undergo additional psychodiagnostic assessment in the MS outpatient center. In this subsample, we confirmed a significant treatment effect of Deprexis on the BDI with a larger effect size (p=0.047; d=0.75). The intervention was safe with regards to adverse events monitored. Worsening of depressive symptoms from below to above the clinical cut-off (BDI > 13) occurred in n=3 patients in the WLC group and n=0 in the Deprexis group. No adverse events were noted with respect to new occurrence of suicidal ideation during the trial in either group.

2.2 Need For A Trial - Rationale

<u>*What is known:*</u>
- Substantial evidence indicates that Deprexis is effective for reducing depressive symptoms in participants without comorbid somatic disorders.
- Preliminary work supports the efficacy of Deprexis to reduce depressive symptoms in MS patients in Germany.
- The literature strongly suggests that therapist-guided internet-based interventions lead to better outcome. We have preliminary evidence that the guided version of Deprexis also leads to larger effects.

<u>*What is not known:*</u>
- Large, definitive trials that could inform clinical practice are lacking for *any* therapeutic approach in MS-associated depression (see AAN guidelines).
- The potential of added email support to enhance efficacy of iCBT in MS is unknown. It is also unknown which patients may benefit most from this added support (e.g. depending on baseline level of depression).
- It is unknown if Deprexis also works in a larger, international, and more heterogeneous MS population.
- Long-term stability of therapeutic effects and strategies to enhance it have not been explored for *any* therapeutic approach in MS-associated depression.

3 Trial Goals

3.1 Primary Endpoint

Our primary hypothesis is as follows: Deprexis is effective for reducing depressive symptoms (as measured by the Beck Depression Inventory – II) at the end of treatment (week 12).

For further details concerning the statistical evaluation please see Chapter 14.3.

3.2 Secondary Endpoints

3.2.1. Secondary endpoints
Secondary endpoints and the secondary hypotheses are listed below.

Quality of life:
- WHO-QoL BREF
- Multiple Sclerosis Impact Scale, MSIS-29

Hypothesis: Deprexis increases quality of life at the end of treatment (week 12)

Fatigue
- Fatigue Scale for Motor and Cognitive Functions (FSMC)
- Chalder Fatigue Scale

Hypothesis: Deprexis decreases fatigue at the end of treatment (week 12)

Depression
- Current diagnosis of MDD according to the M.I.N.I. structured clinical interview (clinician-based rating version 5.0.0)
- Montgomery Asberg Depression Rating Scale (MADRS)

Hypothesis: Deprexis reduces the percentage of patients with a clinical diagnosis of current MDD at the end of treatment (week 12)
Hypothesis: Deprexis reduces the severity of depression as determined by the clinician-rated MADRS at the end of treatment (week 12)

3.2.2. Moderator and safety measures

Neurological impairment

- Patient Determined Disease Steps (PDDS)
- *Expanded Disability Status Scale* (EDSS) (*in selected centers only)*

Cognitive function

- Brief Cognitive Assessment in MS (BICAMS)
- *Ancillary cognitive assessments (*in selected centers only)*
    - *Matrix Reasoning**
    - *COWAT**
    - *Animal Naming**
    - *Oral Trails**
    - *Golden Stroop**
    - *WTAR**
    - *Mini Snellen**
    - *MRR**

Suicidal ideation (safety)

- Suicidal Behaviors Questionnaire-Revised (SBQ-R)

ADDITIONAL MODERATORS *(*in selected centers only)*

- Perceived stress scale (PSS)
- Self-efficacy for managing chronic disease (SES6G)
- Berlin Social Support Scales (BSSS)
- *Experience Questionnaire**
- *Psychological vulnerability questionnaire**
- *Committed Action Questionnaire**
- *Apathy Evaluation Scale**
- *Apathy Scale**
- *Hassles and Uplifts Questionnaire**
- *Cognitive Health Questionnaire**

3.3 Design

Characteristics

Three arm, randomized, controlled, multicenter trial

Groups

- Deprexis
- DeprexisPlus
- Waitlist Control

Study organization

- Multicentric with 5 academic centers recruiting
- Total recruitment of 375 patients

3.4 Schedule

Trial Preparation

- Ethics committee and Competent Authority Approvals approx. Q4/2016
- Construction of the online study platform approx. Q3/2016
- Completion of the therapist manual approx. Q4/2016

Screening and Recruitment

- Q2/2017

Completion of Trial

- Last patient in: approx. Q2/2019
- Last patient out: approx. Q4/2019
- Completion of statistical evaluation and interpretation & presentation of final report: approx. Q2/2020

4 Patient Selection

Our target for recruitment is 375 patients (see Chapter 14.1 for sample size estimations).

4.1 Inclusion criteria

- age $\geq$ 18
- neurologist-confirmed diagnosis of MS
- self-reported depressive symptoms (BDI-Fastscreen $\geq$ 4)
- fluent in German or English (depending on study site)
- willingness to engage in self-administration of an iCBT intervention for 12 weeks and complete follow-up
- ability to travel to the outpatient center for two clinical assessments (baseline and week 12)
- internet access at home
- informed consent by patient

4.2 Exclusion criteria

- unwilling or unable to consent
- diagnosis of bipolar disorder or psychosis (as determined by M.I.N.I structured interview)
- substantial neurocognitive impairments such as dementia or autism
- moderate or high risk of suicide (according to MINI module C) or by clinical impression
- very severe depression that would interfere with the ability to participate in the study (based on clinical judgment by the physician at the recruitment site). Patients with very severe depression will be referred to psychiatric services for immediate treatment.
- current psychotherapy/behavioral treatments for depression
- started pharmacotherapy for depression within the last 2 months
- MS relapse or steroid treatment in the last 4 weeks
- concurrent participation in another interventional clinical trial
- refusal to saving, processing and forwarding of pseudonymized data

5 Treatment

5.1 Description of the treatment

In this trial, we will use Deprexis, either as a stand-alone internet-based intervention (Deprexis) or with added standardized email support by a clinical psychologist (DeprexisPlus, see below). We have recently adapted Deprexis to MS-specific needs. Content regarding psychological challenges frequently facing MS patients was incorporated into the Deprexis format and the new version was piloted in focus groups of patients. In the IDEMS trial, we will only use the MS-adapted version of Deprexis.

MS-specific Deprexis: Deprexis is an online tool based on principles of CBT. It consists of 10 sequential modules plus an introduction and a summary module. Deprexis implements the technique of simulated dialogue by giving the user multiple choice options and tailoring the subsequent content to the patient's responses. Thereby, the user's responses determine the course of each module. Depending on the user's speed, each module can be completed in less than 60 minutes. Contents are (1) psychoeducation, (2) behavioral activation, (3) cognitive modification, (4) mindfulness and acceptance, (5) interpersonal skills, (6) relaxation, physical exercise and lifestyle modification, (7) problem solving, (8) expressive writing and forgiveness, (9) positive psychology, and (10) emotion-focused interventions. The newly developed MS-Deprexis contains several MS-specific elements, the vast majority of them included in the first module: (1) The program now clarifies that it is intended specifically for MS patients; (2) several illness parameters are assessed early on in an interactive sequence, including time since diagnosis, symptom severity, and subjective impairment; (3) an interactive sequence introduces the concept that biological as well as psychosocial factors might contribute to depression in MS; (4) the psychoeducational sequence introducing a cognitive-behavioral model of depression has been modified; for example, users can now reflect on optimistic as well as pessimistic cognitive responses to having MS (e.g., "Having MS makes me appreciate every day even more" vs. "Having MS means the future is bleak and hopeless for me"); (5) the section on subjective reasons for depression has been modified; having MS is now included as a potential reason; and (6) the section in which previous research is discussed has been modified to include the results from our phase II trial in MS patients. In addition to these changes, minor changes have been made to other program modules. For example, the module on activity scheduling now acknowledges that certain activities may be inappropriate because of the MS diagnosis, and users are encouraged to select only activities that they feel safe doing. Piloting, qualitative, and quantitative assessment of the MS-specific Deprexis supported its suitability in this population.

MS-specific DeprexisPlus (guided version): This group will receive the web-based Deprexis program, in its modified version to increase suitability for MS patients, plus scheduled e-mail contact with a therapist. The manual for the e-mail support will be based on a manual developed for the IDEMS trial (see appendix). The manual aims to maximize the e-mail support's suitability for the needs and requirements of MS patients. Experiences from recent projects in which detailed clinician manuals for Deprexis support conditions have been developed will also be considered in the protocol modification (http://www.e-compared.eu/research/trials-design/). The basic structure of the e-mail support will be as follows (based on (25)): At the beginning of the treatment, a therapist will introduce herself (or himself) via e-mail, which will be integrated (secure webmail) into the online study platform. Participants will be informed that they can contact their therapist whenever they want to. Once a week, the therapist will write a short e-mail with feedback based on participants' program usage over the previous week (i.e., as in previous studies with the supported Deprexis version, the therapists will be able access information regarding which modules the participants have engaged with and for how long). This feedback will also acknowledge participants' response to a brief mood measure and to the PHQ-9 (current depression), which they are asked to complete at regular intervals as they work through the program. Progress with the relevant Deprexis related tasks and specific therapeutic techniques, or principles can be briefly discussed in the e-mails, as well as MS-specific questions or concerns. The main function of this feedback is to encourage participants' independent work with the Deprexis program and to enhance engagement. Where there is no online activity by a participant, therapists will offer their help and assistance and will ask if the participant is facing any problem with the program or with the tasks. When participants ask a question, therapists will provide an answer

within 3 days. If a therapist is on leave, participants will be notified there may be a more delay in answering questions. Two therapists with a qualification in clinical psychology will be responsible for email support in the trial (one for all patients enrolled in the German study sites and one for all patients enrolled in the US). Supervision by experienced clinical psychologists will be provided at least monthly for German-speaking and English speaking participants. To ensure consistent quality, and to be able to estimate the extra cost of guided support, all e-mail messages sent by the therapists will be saved (together with the time taken to read patient emails and respond to these) and stored in a secure file. Emails will be discussed in supervision to maintain fidelity and quality during the trial. In addition, at the end of the trial, a random 20% of emails will be rated by an independent trained assessor for treatment fidelity.

Deprexis booster: During the maintenance phase of the trial**,** Deprexis access will remain open for all patients who were randomized to either the Deprexis or the DeprexisPlus group. However, these patients will be re-randomized to receive additional booster session or just continued access to the program without any additional measures. In the booster condition, additional content (on relevant topics such as maintaining treatment gains and preventing relapses and additional modules introducing advanced CBT techniques) will be unlocked. In addition, the participants will receive weekly automated messages encouraging them to work with the program. The participants randomized to no booster will simply have continued access but not see the added content and not receive automated email reminders.

5.1.1 Known Adverse Events

In the previous monocenter trial of Deprexis in MS, we considered new occurrence of suicidal ideation or intent as well as worsening of depressive symptoms above the clinical threshold as potential adverse events. None of the enrolled participants met the predefined criterion for acute risk of suicide (response 3a or 3b on SBQ-R item 3 plus score of 5 or 6 on SBQ-R item 4) at baseline or the after the intervention. For the low threshold definition using BDI item 9, the criterion was not met by any patient in the Deprexis group (pre: n=0; post: n=0) but in one instance in the WLC group (pre: n=1; post: n=1). For SBQ-R item 4, n=2 patients met the low threshold criterion before and n=0 patients after Deprexis in the treatment group. In the WLC group, this criterion was met in n=4 at baseline and n=3 nine weeks after baseline. However, no evidence for new occurrence of suicidal ideation was seen during the trial in either of the groups using the low threshold criteria. Worsening of depressive symptoms during the trial from below to above the cut-off for "caseness" (BDI > 13) was observed in n=3 patient in the WLC but not in the Deprexis group.

5.1.2 Treatment Plan

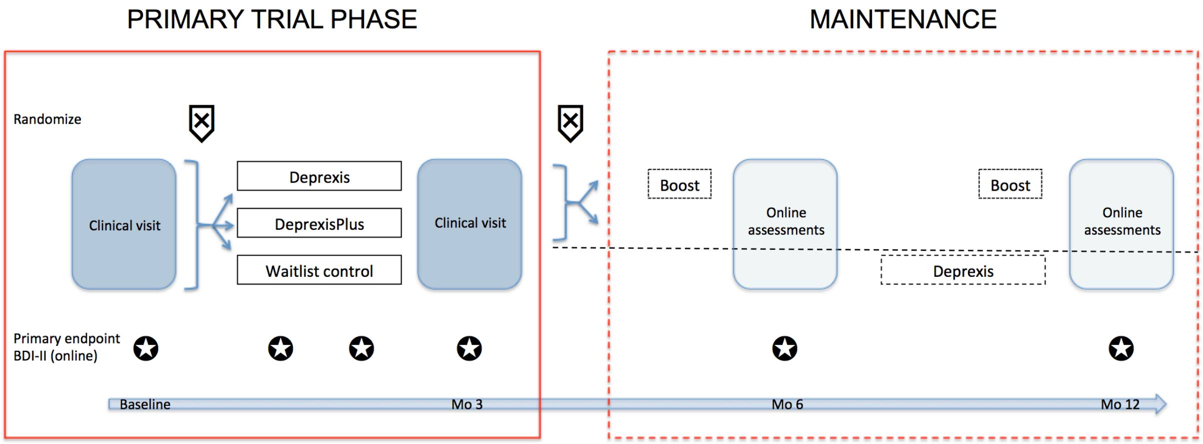

Figure 1: Trial Design Schematic and Treatment Plan.

5.1.3 Compliance

Ascertainment of compliance

We will assess time spent working with the Deprexis program by each patient as a measure of treatment adherence. The Deprexis interface tracks usage time of each participant, thereby allowing evaluation of treatment adherence. The usage log of Deprexis uses 5-minute blocks and excludes each block of inactivity so that the logged usage times is a good estimate of time spent working with the program. We will use the time spent working with the program and the number of modules completed (with at least 15 minutes spent in the module) as an indicator of treatment adherence. For DeprexisPlus, we will also collect data on number of email contacts per patients and total time spent on each email.

5.2 Emergencies

In case of emergency supposedly due to application of IMP treatment is immediately discontinued. Regulatory reporting duties apply. As we consider occurrence of suicidal ideation and intent as a potential adverse event in patients with depressive symptoms, we have developed specific SOPs for reponsing to such events (see SOP suicidality in the appendix).

6 Trial Conduction

6.1 Screening and Recruitment

Screening will happen within the participating centers.

Recruitment

- Recruitment happens as a distinct informational visit.

6.2 Informed Consent

Patients will be informed about the conduction of the trial in a personal conversation by a physician. They will receive the written patient information, and be given time to read it thoroughly and without haste. If further questions arise, the physician will answer them openly and correctly. If the patient is able to consent, but unable to sign the documents, an independent witness may sign the forms to document the orally given consent by the patient.

6.3 Prevention of simultaneous inclusion in multiple trials

Inclusion in another interventional trial is an exclusion criterion. It is the duty of the recruiting investigator to rule out current participation in another trial. Patient information will explicitly state the impossibility to partake if already participating in another trial.

6.4 Enrolment

- Recruitment of new patients at any of the study sites will be tracked in pseudonymized form by the study platform

6.5 Visits, Investigations and deviations of standard-of-care

Methodological Continuity

Same methods to measure variables and outcome parameters will be used throughout the conduction of the trial.

Time points

Treatment will be administered for 12 weeks during the primary trial phase.

Complementary Scientific Program

Several ancillary studies are planned, some of them only in selected sites:

Neuroimaging: Structural and functional (resting state and emotion regulation task) MRI studies are planned at several sites. These will be conducted only during the clinical visits (V1 and V2).

Psychological moderators and predictors of treatment response: Additional paper and pencil questionnaire to measure potential psychological moderators and treatment reponse predictors will be obtained at several sites. These will be conducted only during the clinical visits (V1 and V2).

Molecular and cellular shifts in the immune system: Obtaining blood samples for storage of serum, plasma and cryopreserved peripheral blood mononuclear cells (PBMCs) are planned at several sites. These materials will be used to explore potential immunological markers of treatment response. Blood samples will be obtained only during the clinical visits (V1 and V2).

Investigation	V1 (wk 0)	Interim (wk 4)	Interim (wk 8)	V2 (wk 12)	V3 Follow-up (M 6)	V4 Follow-up (M 12)
Check for selection criteria	X					
Informed Consent	X					
Case history	X					
Clinical Examination	X			X		
Questionnaires	X	X	X	X	X	X
Demographic data	X					
Laboratory examination						
Blood sampling (optional)	X			X		
MRI (optional)	X			X		
Incidence of AEs/SAEs		X	X	X	X	X

Blue: Clinical visits at the study site; **Black**: Online assessments

In case of a loss to follow up we contact the local registration authorities to ensure we can reach the highest amount of patients possible, even if they moved.

6.5.1 V1 (clinical visit)

Informed consent is obtained and selection criteria will be checked. If all inclusion criteria are met and all exclusion criteria are excluded baseline examination will be performed and patients will be randomized.

The following data will be collected:

- Case history and demographic data
- Clinical examination, including
  - a basic medical examination
  - a neurological examination
  - a structured clinical interview for psychiatric symptoms
  - self-report questionnaires
- *Laboratory examination (optional)*
  - *Immunology*
- *Neuroimaging (optional)*
  - *Structural MRI*
  - *Resting-state fMRI*
  - *Task fMRI*

6.5.2 V2 (clinical visit) at 12 weeks post randomization (+/- 7 days permissible)

Visits will be conducted on site at unit/neurological ward:

- Clinical examination, including
  - a basic medical examination
  - a neurological examination
  - a structured clinical interview for psychiatric symptoms
  - self-report questionnaires
- Incidence of AEs
- *Laboratory examination (optional)*
  - *Immunology*
- *Neuroimaging (optional)*
  - *Structural MRI*
  - *Resting-state fMRI*
  - *Task fMRI*

6.5.3 Follow-Up assessments – V3 and V4 (online) (+ 28 days permissible)

Follow-up assessments will be conducted online. The following data will be collected at V3 and V4:

- Self report questionnaires
- Incidence of AEs

6.5.4 Laboratory Details

No routine laboratory markers will be obtained. In some centers, ancillary scientific studies are carried out to study immunological correlates of depressive symptoms in MS and their response to treatment using serum samples and PBMCs (see above)

6.6 Duration of trial participation for the individual subject

Prerequisites of trial completion for the individual subject

- Duration of treatment: 12 weeks
- Duration of follow-up: 1 year after study inclusion

7 Risk-Benefit-Consideration

Risks, Adverse Reactions, Burden, Advantages and Disadvantages for Participants

RISKS

Risk of the treatment: The intervention is generally considered low risk as Deprexis has been used in numerous clinical trials without evidence for adverse events and is categorized as a “low risk” medical device according to its German CE®-certification. The FDA has reviewed Deprexis in July 2015 and classified it as a mobile medical application. The FDA does not intent to enforce any regulatory requirements under applicable provisions of the Federal Food Drug and Cosmetic Act, Section 513(g). A potential safety concern regarding an online intervention targeted at depression is suicidal ideation and intent.

Procedures implemented to control risk: Acute risk for suicide at baseline (as determined by the SBQ-R, see above) will lead to exclusion from the trial and patients will immediately be referred to specialists for crisis intervention. In our previous trial (26) we found no evidence for the new occurrence of suicidal thoughts during the trial or at 6-months follow-up in the Deprexis or waitlist control group. Similarly, previous trials of Deprexis (including the recent trial of patients with severe depression (23)) also showed no evidence for increased suicidal ideation and intent. However, in the proposed trial we will establish a detailed protocol for detection and response to suicide risk. During the clinical visits, all patients will be asked about suicidal ideation and intent. In addition, we will obtain information about emergency contacts (friends/family) in a standardized manner. If participants express suicidality during their clinical visit, the site PI (neurologist or experienced clinical psychologist) will be contacted immediately by the study coordinator and will explore things further with them in person. If participants appear to present an immediate danger to themselves, then standard procedures established at each site will be followed for possible commitment. Participants will be informed in the informed consent form about the possibility that confidentiality may need to be breached if they express an immediate, serious danger to themselves. Finally, the secure online study platform will include a feature where helpline numbers will be automatically displayed if response on the BDI-II indicates suicide risk at any time (item 9 = 3). In this case, an automated email alert will also be sent to the study center of the patient for a follow-up phone call by the study coordinator and the site PI (neurologist or experienced clinical psychologist).

Risk of blood sampling: Blood sampling is considered low risk. Blood drawing from a vein may cause pain from insertion of the needle, light headedness, faintness, bruising, localized bleeding which may

look and feel like a bruise, and rarely inflammation of the vein, clotting of the vein and/or infection at the needle site.
Procedures implemented to control risk: Blood sampling will be conducted according to established protocols and performed by authorized personnel (study nurse or physician).

Risk of MRI examinations: There are no specific side effects from having an MRI scan, although some patients become claustrophobic (fear of enclosed space) during the MRI scan. Because the MRI uses magnets, participants with cardiac pacemakers, certain artificial heart valves, and/or other metal-lic/electronic material in their bodies cannot undergo MRI imaging and will not be eligible for this study. MRI is painless and requires only that you lie in the scanning machine. The machine produces loud sounds. It is therefore essential that you wear earplugs during the MRI scan to protect your hear-ing. Patients with severe claustrophobia during prior MRI scans or patients with a prior allergic reac-tion to gadolinium should not participate in this study.
Procedures implemented to control risk: Patients will carefully be examined for potential contraindica-tions for MRI scans and scans performed by authorized personnel under appropriate supervision of a neuroradiologist.

BURDEN

The main burden for participants in the trial is coming in for the clinical examinations (V1 and V2), completing the online questionnaires and working with the Deprexis program. Patient will be reim-bursed for their time and effort.

ADVANTAGES

Participants will receive a novel online program to reduce depressive symptoms free of charge, either immediately or after waiting for 6 months (i.e. all patients will receive treatment). Participants may also benefit from a throrough clinical and psychological examination conducted at V1 and V2 by au-thorized personnel under the supervision of experienced clinicians.

8 Discontinuation and Ongoing Treatment

8.1 Premature discontinuation of participation for an individual subject

Reasons to discontinue participation (Criteria of discontinuation)

The following conditions are obligatory reasons to discontinue further participation in the trial accord-ing to the protocol:

- Decision of the patient
- Any further situation rendering further participation potentially harmful to the patient at the discretion of the investigator

The following conditions are possible reasons to discontinue further participation in the trial according to the protocol, and have to be reviewed as soon as possible by the principal investigator:

- Serious adverse event (SAE) and Suspected unexpected serious adverse reaction (SUSAR)
- Retrospectively assessed exclusion criterion

8.2 Premature discontinuation of the trial

A premature discontinuation of the trial may be decided if new scientific data emerging during the course of the trial changes the risk-benefit-balance significantly. If such data emerges, recruitment and treatment of currently treated patients will be paused immediately. A final decision on continuation or termination of the trial will then be made by the principal investigator.

SAEs will be assessed in detail at the second clinical visit for each patient with a specific focus on new occurrence of psychopathology and suicidal ideation and intent (based on MINI structured interview and the SBQ-R at the M3 clinical visit). There are no predefined stopping rules.

8.3 Ongoing procedures besides the protocol

After premature discontinuation of the trial

Patients will be followed up at the same time points as lined out in the regular study protocol for safety endpoints if they agree to.

After regular completion of the trial

Patients may seek advice on the study or related issues after completion of the trial, but may be then referred to structures of regular health care (GPs, neurologists, psychiatrists, outpatient clinics, etc.) in case the scope of the issue is covered by standard of care.

9 Adverse Events

The following chapter contains the definitions of and procedures to assess and grade adverse events. Furthermore, it states the chain of report for severe or unexpected events.

9.1 Definitions

Adverse Event - AE

Any untoward medical or psychological occurrence temporally associated with the use of the intervention, but not necessarily causally related.

Every AE has to be rated concerning its severity:

- Mild: The AE is transient and easily bearable for the patient.
- Moderate: The AE causes inconveniences to the patient and interferes with his or her usual activities.
- Severe: The AE causes significant disturbances for the patient‘s usual activities.
- whether criteria of an SAE are fulfilled

Every AE has to be judged in terms of causal relation to the IMP:

- no causal relation
  The event is well understood and derives from another cause.
- possible causal relation
  An event with an understandable temporal relation to IMP application, well-fitting to a known pattern of reaction to the IMP, but easily attributable to several other factors.
- probable causal relation
  An event with an understandable temporal relation to IMP application, well-fitting to a known or expected pattern of reaction to the IMP. The event resolves after cessation of IMP application and is not explicable through other known factors of the patient’s clinical condition.
- certain causal relation
  An event with an understandable temporal relation to IMP application, well-fitting to a known or expected pattern of reaction to the IMP. The event resolves after cessation of IMP application.

Documentation on MS relapse or progression and hospitalization due to MS relapse or progression should be made on the designated CRF-pages.

Serious Adverse Event - SAE

Every AE fulfilling any of the following criteria is considered an SAE:

- Suicidal ideation or intent (as measured by a score of 3 on BDI-II item 9 at any assessment, during the clinical interview at V2 or spontaneous report in a web message to the therapist or by contacting the study site)
- Hospitalization due to psychiatric disorder classified according to ICD10 or DSM5
- Lethal or life-threatening (incl. suicide or suicide attempt)

Suspected Unexpected Serious Adverse Reaction - SUSAR

SUSARs are suspected unexpected serious adverse events, causally related to the application of the intervention. The following criteria have to be fulfilled:

- Type or severity of the event are not in accordance with the available information on the intervention.
- Lethal or life-threatening (incl. suicide or suicide attempt)

9.2 Documentation of AEs and SAEs

Every AE has to be documented regardless of causal relationship. Documentation contains kind of event, start and duration, intensity and causal relationship. Related symptoms, clinical and laboratory findings should be summarized to a single AE. A sheet to document AEs is part of the CRF; furthermore, SAEs have to be separately documented on a distinct SAE sheet. If the necessary information is currently not available, follow-up reports have to be completed and transferred. In case of a lethal event, the autopsy report should ideally be included.

9.3 Chain of report for SAEs and SUSARs

Obligations and deadlines given by the authorities remain untouched. This chapter is solely for the investigator’s information.

Table: Reports from Investigator to Principal Investigator		
Kind	Deadline	
AE	Upon completion of patient follow-up	Written report in CRF
SAE	Within 15 days	Written report in CRF and on separate, distinct SAE form

Kind	Deadline	Investigators
SAE	On Request	
SUSAR	Case report within 15 days	x
SUSAR (Death)<br><br>Follow-Up-Report (if initially incomplete)	Case report within 7 days<br><br>After 8 days	x<br><br>x

<u>Exceptions</u>
The following SAEs should be <u>excluded of the report chain</u> in the course of this trial:

- SAEs occurring after trial inclusion but before treatment initiation
- Events with hospitalization planned before the inclusion to the trial

<u>Principal Investigator Reporting Duties</u>

The principal investigator documents every reported SAE. The principal investigator furthermore reports every SUSAR immediately, in any case within 15 days after it became apparent, to the participating investigators. A lethal or life-threatening SUSAR is reported immediately, in any case within 7 days after it became apparent, to the participating investigators. A follow-up report within further 8 days can be transmitted if information retrieved was initially incomplete.
All data will be transformed in pseudonymized form.

9.4 Data Monitoring Committee (DMC)

The DMC is an independent committee regularly assessing safety data during the course of this study. Primary interest of the DMC is the safety of trial participants and integrity and validity of collected data. The DMC reviews frequency and severity of SAEs. It states a recommendation to the principal investigator whether to continue or discontinue trial conduction. Details on the predefined criteria are given in Chapter 12.5.3.

Members of the DMC are
Sarah Minden, M.D., Brigham and Women’s Hospital, Harvard Medical School, Boston, USA
Carsten Spitzer, M.D., Asklepios Clinic Tiefenbrunn, Germany

10 Documentation

10.1 Case Report Form (CRF)

Acquired data is documented in paper CRF as well as eCRFs (IDEMS study platform). A copy of the paper CRF can be found in the appendix.

10.2 Investigator Site File (ISF)

All essential documents according to ICH GCP Chapter 8 are filed in the Investigator Site File on site.

10.3 Trial Master File (TMF)

All essential documents according to ICH GCP Chapter 8 are filed in the Trial Master File at the coordinating centre (NCRC).

11 Quality Management

11.1 Assessment of trial conduction and data quality

Indicators of quality for trial conduction are

- Adherence to recruitment rate
- Adherence to selection criteria
- Adherence to per-protocol-treatment
- Adherence to visit schedule

11.1.1 Monitoring

Internal Monitoring for German sites will be conducted by the coordinating centre / principal investigator. Every patient will be monitored for selection criteria and informed consent. Internal monitoring will also verify if appropriate SOPs were followed in case an SAE (suicidal ideation or intent) occurred. The investigator assures complete and unrestricted access to study data for the monitor. Monitoring details will be fixed in a monitoring manual.

Most of the data will be directly entered to the electronic study platform (mostly be the participant) or CRF and are thus considered source data.

11.1.2 Audits / Inspections

No internal audits are planned, but the Principal Investigator reserves the right to initiate a previously not planned audit.

11.2 Standardization and Validity

All rating scales have previously been standardized and validated.

12 Data Management

All personalized data is acquired in a pseudonymized manner. Every patient is attributed a unique ID in the course of inclusion. The investigator keeps a confident list containing full patient name and attributed ID. This list is accessible only to the local study team and monitors. Source data files are accessible to monitors, auditors and inspectors.

12.1 Data Acquisition and CRFs

Acquisition is performed using *paper Case Report Forms (CRF),* distributed by the sponsor. Additional data are acquired in the study platform.
The original is intended for the principal investigator/coordinating study center, a copy remains on the local site. Forms have to be completed with a pen, use of pencils is not permitted. Corrections have to

be done as follows: any mistake is crossed out by a single line, correct information is written next to it, dated and signed by the investigator and ideally a reason for correction is given. If missing information prohibits completion of a field, an explanation should be given.

12.2 Data Processing

For paper CRFs, the principal investigator/coordinating centre transposes data to a digital format. Data will be checked for range, validity and consistency. Implausible or missing data can be corrected in accordance with the investigator, documentation of correction is stored along with the CRFs. Validated data is stored in a database. At the end of the trial official database closure is documented. For evaluation of data we intend to use the current version of SPSS.

12.3 Creation of Pseudonym

Patients will be incrementally numbered based on date and time of inclusion. The number consists of a letter referring to the site of inclusion (B=Berlin, H=Hamburg, P=PennState, K=Kansas City, L=Los Angeles) and four patient-related digits (i.e. the first patient in Berlin will receive the code “B0001”).

12.4 Randomization

The trial will use a central, web-based randomization tool built into the study platform. Randomization will be conducted through this online system, ensuring concealed allocation. Patients will be randomized 1:1:1 to one of the three trial arms (no blocking, no stratification). To ensure concealed allocation, eligibility will be determined and all baseline assessments completed before randomization in compliance with CONSORT guidelines (extended CONSORT Statement to Randomized Trials of Non-pharmacological Treatment (27) and CONSORT Statement for eHealth (28)). Group assignment will be communicated automatically by a webmessage in the study platform. The clinicians and raters who will be conducting clinical assessments (structured interviews) will be blind to treatment assignment (single blind RCT).

Sample size estimation

A sample size of 100 patients per intervention group gives a conjunctive power (probability of rejecting both null hypotheses comparing Deprexis and DeprexisPlus to waitlist control) of 90% for a Dunnett test at the usual one-sided significance level of 2.5% assuming standardized mean differences of 0.5 for Deprexis vs. WLC and 0.8 for DeprexisPlus vs. WLC in the primary outcome change in BDI from baseline to week 12. Adjusting for 20% dropout we aim to recruit 125 patients per group resulting in a total sample size of 375 patients. The power was simulated with 10,000 replications using EAST 6.3.

12.5 Statistical Evaluation

Statistical analysis will be conducted in accordance with the following guidelines of the International Conference on Harmonization (ICH):

- ICH E3: Structure and Contents of Clinical Study Reports
- ICH E6: Good Clinical Practice (GCP). Consolidated Guideline
- ICH E9: Note for Guidance on Statistical Principles in Clinical Trials

12.5.1 Hypotheses

Primary hypothesis: All analyses will be conducted as intention-to-treat (ITT). To definitively test the effectiveness of Deprexis for reducing depressive symptoms in MS at the end of treatment, the primary outcome change in BDI from baseline to week 12 will be will be analyzed by means of linear mixed effect models for repeated measures adjusted for baseline measurements with fixed effects for intervention, center, time and baseline BDI score, and random subject effects for individual patients including all patients with at least one post-baseline measurement (29). Least squares means will be reported for the intervention groups with 95% confidence interval (CI) as well as the difference between the least squares group means with 95% CI. Deprexis vs. WLC and DepresixPlus vs. WLC will be tested by a Dunnett test controlling the familywise type I error rate at the level of 2.5%

(one-sided). *The primary hypothesis will be tested in a confirmatory manner.* In a secondary step, we will determine the added value of therapist email support Deprexis vs. DeprexisPlus at a two-sided level of 5%, if efficacy of Deprexis and DeprexisPlus for reducing depressive symptoms in MS could be demonstrated.

Secondary hypotheses: The analyses of secondary and tertiary endpoints (such as BDI-FS, QoL, cognitive function and fatigue scales) will follow the same approach as the analyses described for the primary endpoint. The proposed trial will create a large data set from n=400 MS patients and followed for 12 months that will enable us to gain valuable insight into the potential predictors, confounders, intermediates in the causal pathway, and interactions of Deprexis(Plus) treatment effects. In supporting exploratory analyses we will investigate potential moderators of treatment effects by including these and their interaction with treatment in the linear mixed effects models described above. Potential moderators include disease-modifying therapies (DMTs), level of disability at baseline (as measured by Disease Steps and patient-rated EDSS), cognitive status at baseline (as measured by BICAMS), and level of fatigue at baseline (as measured by the FSMC).

12.5.2 Evaluation of primary endpoint

Primary hypothesis is tested in a confirmatory manner:
All analyses will be conducted as intention-to-treat (ITT). The specific statistical analyses for each aim are as follows: **Aim 1:** To definitively test the effectiveness of Deprexis for reducing depressive symptoms in MS at the end of treatment, the primary outcome change in BDI from baseline to week 12 will be will be analyzed by means of linear mixed effect models for repeated measures adjusted for baseline measurements with fixed effects for intervention, center, time and baseline BDI score, and random subject effects for individual patients including all patients with at least one post-baseline measurement (29). Least squares means will be reported for the intervention groups with 95% confidence interval (CI) as well as the difference between the least squares group means with 95% CI. Deprexis vs. WLC and DepresixPlus vs. WLC will be tested by a Dunnett test controlling the familywise type I error rate at the level of 2.5% (one-sided). **Aim 2:** To determine the added value of therapist email support Deprexis vs. DeprexisPlus will be tested at a two-sided level of 5%, if efficacy of Deprexis and DeprexisPlus for reducing depressive symptoms in MS could be demonstrated. **Aim 3:** To explore the long-term stability of therapeutic effects (12 months) and the potential of a booster session to enhance maintenance, we will conduct similar linear mixed effect models for repeated measures in a two group comparison (Booster condition vs No Booster) during the extension phase (i.e. from week 12 (V2) to the end of the trial (month 12) accounting for the intervention (Deprexis or DeprexisPlus). Here, we will only include patients who had originally been randomized to either Deprexis or DeprexisPlus in primary trial phase.
Missing data: Although the mixed models described above are robust to a certain extent to missing data, sensitivity analyses will be performed as supporting analyses, if missing data are substantial and suspected to be due to dropout. Models that can account for informative dropout such as shared random effects models will be employed to explore the sensitivity of the analyses to certain dropout mechanisms (30). Standard procedures for reporting of adverse events will be used. Adverse events will be summarized as frequencies and percentages by intervention group. No interim analyses are foreseen. The ITT population as well as all other details will be defined in the Statistical Analysis Plan, which will be finalized before database lock.

12.5.3 Interim analyses by the DMC

No interim analyses are planned.

13 Reporting

13.1 Biometric Report

Statistic evaluation and writing of a report is performed by the biostatistician Prof. Dr. Tim Friede, Universitätsmedizin Göttingen, in close collaboration with the principal investigator. The content of this report is confidential.

13.2 Final Report

The final report of the trial follows the requirements of the guideline ICH E3: Structure and Contents of Clinical Study Reports. After completion of the biometric report, the prinicipal investigator writes the final report.

13.3 Publication

Results of the trial will be published regardless of the results. Any publication has to be agreed upon as determined in the Academic Collaboration Agreement in its fully executed form.

14 Ethical, juridical and administrational issues

14.1 Juridical prerequisites for trial initiation

The principal investigator and every participating investigator agree to comply to the GCP standards and national regulations.

Ethics Committee Vote

Study protocol, patient information and consenting form are presented to the Ethics Committee for evaluation at each study site. A positive vote is mandatory for trial initiation.

Patient information and Informed Consent

Patient information

Previous to trial inclusion, every patient will be informed about purpose, possible risks and benefits by the local investigator orally and in writing.

Informed Consent to Participation

Every patient consents in written form to participate in the trial. The patient has to be provided with enough time to think about the trial, ask possibly remaining questions and form a decision. Consent forms are signed and individually dated by patients and the investigator. If the patient is currently not able to write and sign, testified oral consent is acceptable. Information and consent form are made out in duplicate, one copy remains with the patient, the original with the investigator.

Insurance for Participants

All sites are responsible to ensure that they have sufficient liability insurance for conducting this study. No further participant insurance beyond the liability insurance of the participating sites has been obtained.

Privacy

Every participant will be informed about the storage, evaluation and publication of their medical data in a pseudonymized manner. Patients have the right to be informed about the saved data. Patients disagreeing cannot participate in this trial.

14.2 Storage and Access

Original study files, including CRFs, will be stored by the coordinating centre for at least 10 years after trial completion. The investigator stores every administrative documents, as well as signed consent forms, CRF copies and the general trial documentation for the same period of time.
Archiving of source data is left to the discretion of the local investigator, but has to last at least 10 years. Patient ID logs have to be archived for 15 years according to the 2001/83/EG guideline.

15 Bibliography

1. Siegert RJ, Abernethy DA (2005): Depression in multiple sclerosis: a review. *Journal of neurology, neurosurgery, and psychiatry*. 76:469-475.
2. Viner R, Fiest KM, Bulloch AG, Williams JV, Lavorato DH, Berzins S, et al. (2014): Point prevalence and correlates of depression in a national community sample with multiple sclerosis. *Gen Hosp Psychiatry*. 36:352-354.
3. Patten SB, Beck CA, Williams JV, Barbui C, Metz LM (2003): Major depression in multiple sclerosis: a population-based perspective. *Neurology*. 61:1524-1527.
4. Feinstein A, Magalhaes S, Richard JF, Audet B, Moore C (2014): The link between multiple sclerosis and depression. *Nat Rev Neurol*. 10:507-517.
5. Hakim EA, Bakheit AM, Bryant TN, Roberts MW, McIntosh-Michaelis SA, Spackman AJ, et al. (2000): The social impact of multiple sclerosis--a study of 305 patients and their relatives. *Disability and rehabilitation*. 22:288-293.
6. Jose Sa M (2008): Psychological aspects of multiple sclerosis. *Clinical neurology and neurosurgery*. 110:868-877.
7. Tarrants M, Oleen-Burkey M, Castelli-Haley J, Lage MJ (2011): The impact of comorbid depression on adherence to therapy for multiple sclerosis. *Multiple sclerosis international*. 2011:271321.
8. Feinstein A (1997): Multiple sclerosis, depression, and suicide. *Bmj*. 315:691-692.
9. Arnett PA, Randolph JJ (2006): Longitudinal course of depression symptoms in multiple sclerosis. *J Neurol Neurosurg Psychiatry*. 77:606-610.
10. Ensari I, Motl RW, McAuley E, Mullen SP, Feinstein A (2013): Patterns and predictors of naturally occurring change in depressive symptoms over a 30-month period in multiple sclerosis. *Multiple sclerosis*.
11. Goldman Consensus G (2005): The Goldman Consensus statement on depression in multiple sclerosis. *Multiple sclerosis*. 11:328-337.
12. Ehde DM, Kraft GH, Chwastiak L, Sullivan MD, Gibbons LE, Bombardier CH, et al. (2008): Efficacy of paroxetine in treating major depressive disorder in persons with multiple sclerosis. *General hospital psychiatry*. 30:40-48.
13. Schiffer RB, Wineman NM (1990): Antidepressant pharmacotherapy of depression associated with multiple sclerosis. *The American journal of psychiatry*. 147:1493-1497.
14. Koch MW, Glazenborg A, Uyttenboogaart M, Mostert J, De Keyser J (2011): Pharmacologic treatment of depression in multiple sclerosis. *The Cochrane database of systematic reviews*.CD007295.
15. Hind D, Cotter J, Thake A, Bradburn M, Cooper C, Isaac C, et al. (2014): Cognitive behavioural therapy for the treatment of depression in people with multiple sclerosis: a systematic review and meta-analysis. *BMC psychiatry*. 14:5.
16. Mohr DC, Hart SL, Julian L, Catledge C, Honos-Webb L, Vella L, et al. (2005): Telephone-administered psychotherapy for depression. *Arch Gen Psychiatry*. 62:1007-1014.
17. Mohr DC, Likosky W, Bertagnolli A, Goodkin DE, Van Der Wende J, Dwyer P, et al. (2000): Telephone-administered cognitive-behavioral therapy for the treatment of depressive symptoms in multiple sclerosis. *J Consult Clin Psychol*. 68:356-361.
18. Minden SL, Feinstein A, Kalb RC, Miller D, Mohr DC, Patten SB, et al. (2014): Evidence-based guideline: assessment and management of psychiatric disorders in individuals with MS: report of the Guideline Development Subcommittee of the American Academy of Neurology. *Neurology*. 82:174-181.
19. Richards D, Richardson T (2012): Computer-based psychological treatments for depression: a systematic review and meta-analysis. *Clin Psychol Rev*. 32:329-342.
20. Meyer B, Berger T, Caspar F, Beevers CG, Andersson G, Weiss M (2009): Effectiveness of a novel integrative online treatment for depression (Deprexis): randomized controlled trial. *Journal of medical Internet research*. 11:e15.
21. Moritz S, Schilling L, Hauschildt M, Schroder J, Treszl A (2012): A randomized controlled trial of internet-based therapy in depression. *Behav Res Ther*. 50:513-521.

22. Schroder J, Bruckner K, Fischer A, Lindenau M, Kother U, Vettorazzi E, et al. (2014): Efficacy of a psychological online intervention for depression in people with epilepsy: A randomized controlled trial. *Epilepsia*. 55:2069-2076.
23. Meyer B, BIerbrodt J, Schroder J, Berger T, Beevers CG, Weiss M, et al. (2015): Effects of an Internet intervention (Deprexis) on severe depression symptoms: Randomized controlled trial. *Internet Interventions*. 2:48-59.
24. Johansson R, Andersson G (2012): Internet-based psychological treatments for depression. *Expert Rev Neurother*. 12:861-869; quiz 870.
25. Berger T, Hammerli K, Gubser N, Andersson G, Caspar F (2011): Internet-based treatment of depression: a randomized controlled trial comparing guided with unguided self-help. *Cognitive behaviour therapy*. 40:251-266.
26. Fischer A, Schroder J, Vettorazzi E, Poettgen J, Lau S, Heesen C, et al. (2015): An online programme to reduce depression in multiple sclerosis: a randomised controlled trial. *Lancet Psychiatry*.Epub ahead of print Feb 4, 2015.
27. Boutron I, Moher D, Altman DG, Schulz KF, Ravaud P, Group C (2008): Extending the CONSORT statement to randomized trials of nonpharmacologic treatment: explanation and elaboration. *Annals of internal medicine*. 148:295-309.
28. Eysenbach G (2013): CONSORT-EHEALTH: implementation of a checklist for authors and editors to improve reporting of web-based and mobile randomized controlled trials. *Studies in health technology and informatics*. 192:657-661.
29. Mallinckrodt CH, Clark SW, Carroll RJ, Molenbergh G (2003): Assessing response profiles from incomplete longitudinal clinical trial data under regulatory considerations. *Journal of biopharmaceutical statistics*. 13:179-190.
30. Henderson R, Diggle P, Dobson A (2000): Joint modelling of longitudinal measurements and event time data. *Biostatistics*. 1:465-480.
